# Network Reconstruction Reveals that Valproic Acid Activates Neurogenic Transcriptional Programs in Adult Brain Following Traumatic Injury

**DOI:** 10.1007/s11095-017-2130-6

**Published:** 2017-03-07

**Authors:** Gerald A. Higgins, Patrick Georgoff, Vahagn Nikolian, Ari Allyn-Feuer, Brian Pauls, Richard Higgins, Brian D. Athey, Hasan E. Alam

**Affiliations:** 1 Department of Computational Medicine and Bioinformatics, University of Michigan Medical School, Ann Arbor, Michigan USA; 2 Department of Surgery, University of Michigan Medical School, Ann Arbor, Michigan USA; 3 College of Pharmacy, University of Michigan, Ann Arbor, Michigan USA; 4 Department of Computer Science, University of Maryland, College Park, Maryland USA; 5 Michigan Institute for Data Science (MIDAS), Ann Arbor, Michigan USA

**Keywords:** bioinformatics, network modeling, pharmacoepigenomics

## Abstract

**Objectives:**

To determine the mechanism of action of valproic acid (VPA) in the adult central nervous system (CNS) following traumatic brain injury (TBI) and hemorrhagic shock (HS).

**Methods:**

Data were analyzed from different sources, including experiments in a porcine model, data from postmortem human brain, published studies, public and commercial databases.

**Results:**

The transcriptional program in the CNS following TBI, HS, and VPA treatment includes activation of regulatory pathways that enhance neurogenesis and suppress gliogenesis. Genes which encode the transcription factors (TFs) that specify neuronal cell fate, including *MEF2D, MYT1L, NEUROD1, PAX6* and *TBR1,* and their target genes, are induced by VPA. VPA represses genes responsible for oligodendrogenesis, maintenance of white matter, T-cell activation, angiogenesis, and endothelial cell proliferation, adhesion and chemotaxis. NEUROD1 has regulatory interactions with 38% of the genes regulated by VPA in a swine model of TBI and HS in adult brain. Hi-C spatial mapping of a VPA pharmacogenomic SNP in the *GRIN2B* gene shows it is part of a transcriptional hub that contacts 12 genes that mediate chromatin-mediated neurogenesis and neuroplasticity.

**Conclusions:**

Following TBI and HS, this study shows that VPA administration acts in the adult brain through differential activation of TFs responsible for neurogenesis, genes responsible for neuroplasticity, and repression of TFs that specify oligodendrocyte cell fate, endothelial cell chemotaxis and angiogenesis.

Short title: Mechanism of action of valproic acid in traumatic brain injury

**Electronic supplementary material:**

The online version of this article (doi:10.1007/s11095-017-2130-6) contains supplementary material, which is available to authorized users.

## Introduction

The mechanism of action of VPA as an anticonvulsant, mood stabilizer and analgesic in the human central nervous system (CNS) has not been adequately characterized. VPA and its derivatives exhibit a variety of effects which are cell- and tissue-specific, differ based on disease state, age, gender and ethnicity, and may be effective or deleterious. VPA is a weak blocker of sodium and calcium ion channels, and may inhibit key enzymes in the catabolism of gamma-aminobutyric acid (GABA), including ABAT (4-aminobutyrate aminotransferase) at physiologically relevant concentrations (1). There is also evidence that VPA exerts its antiepileptic action through differential regulation of the *GRIN2B* (Glutamate Ionotropic Receptor NMDA Type Subunit 2B) gene (2). VPA is an effective inhibitor of histone deacetylases (HDACs), with an IC_50_ (0.4 mM) well within the therapeutic range of VPA (0.35–0.7 mM in serum). VPA causes robust chromatin decondensation, with potent acetylation of core histones such as H3 and H4 that leads to activation of development gene expression (3,4). VPA has been shown to be neuroprotective in animal models of traumatic brain injury (TBI) (5,6), spinal cord injury (7), and in neurodegenerative disease (8). In a swine model of TBI and hemorrhagic shock (HS) VPA decreases brain lesion size, improves neurologic recovery, and down-regulates genes associated with necrosis, apoptosis, and inflammation (9). The HDAC inhibitor, sodium butyrate, with a mechanism of action like that of VPA, has been shown to activate neurogenesis in rodent brain following ischemic injury (10). These results suggest different ways in which VPA may act in parallel to provide benefit in epilepsy, mood disorders, migraine, as well as recovery following trauma.

It appears that VPA-induced histone acetylation is not sufficient for chromatin decondensation, but rather a downstream effect of HDAC inhibition, suggesting that the drug suppresses the expression of proteins involved in maintenance of heterochromatin and/or uses chromatin remodeling proteins as intermediaries (11–13). It is also routinely used to remove histone methylation in cellular domains such as the lamina-associating domain (LAD) located just interior of the nuclear membrane, a region containing heterochromatin, in which exposure to VPA abolishes H3K27 and H3K9 methylation (14).

Recent studies of the epigenomic control of gene expression that has identified distinct mechanisms through which chromatin interactions mediate transcriptional programs involving topologically-associating domains (TADs), enhancer-promoter loops and actively transcribed regions of the human genome characterized by the histone mark H3K27ac (15–17). It has been shown that SNPs which disrupt the boundaries of TADs cause serious health problems (18).; and causal SNPs exhibit significant allele bias in open chromatin (19). In addition, the greater the number of spatial connections a given enhancer or promoter maintains genomewide indicates both the potency of the regulatory element and its validity (20), especially for CNS genes that are involved in coordinated transcriptional programs of neurogenesis and neuroplasticity (20).

Our hypothesis is that VPA acts through transcriptional activation and repression of specific genes resulting in chromatin-mediated neurogenesis and neuroplasticity in the adult CNS and inhibition of glial scarring. To determine the validity of this premise, we analyzed experimental and public data. We then reconstructed a gene regulatory network that mediates VPA’s mechanism of action in human brain, and found that the drug exerts widespread effects in adult brain including a transcriptional program of neurogenesis and neuroplasticity, involving TFs that have been shown to program neuronal cell fate commitment and suppress oligodendrocyte cell fate.

## METHODS

### Data

**Fig. 1:**
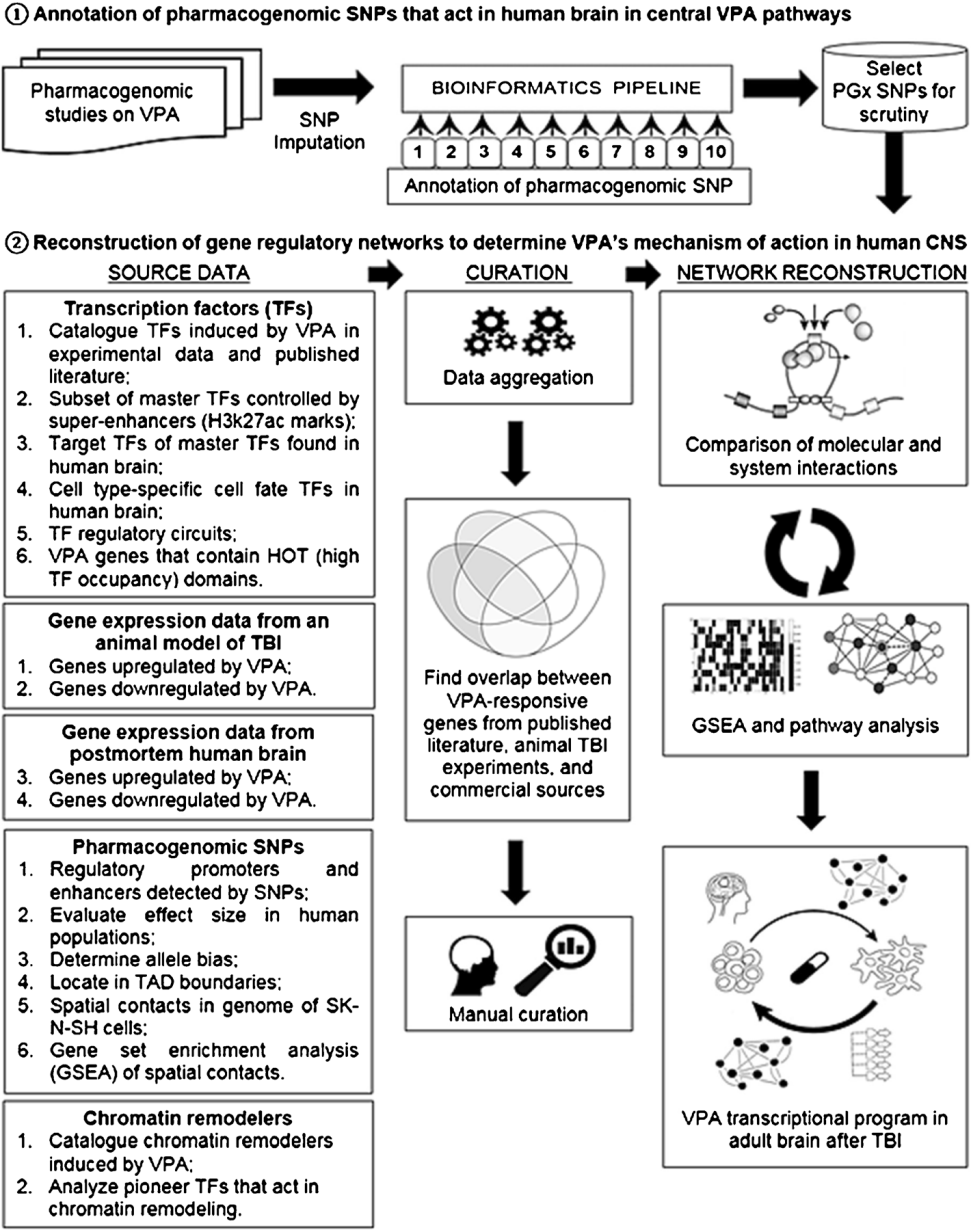
Overview of the experimental design used in this study. **(1)** Shows steps to annotate putative VPA pharmacogenomics SNPS which were **(2)** mechanistically analyzed using integrative bioinformatics and regulatory network analysis methods. In **(1)** numbers refer to the different annotation steps of the bioinformatics pipeline. Details can be found in the text and in Supplement.

Figure 1 shows an overview of the experimental design. Public databases and sources used in this analysis are specified in Supplementary Table 1. These included experimental results from a swine model of TBI and hemorrhage (5,9). Other primary data included microarray expression data from postmortem human brain tissue obtained from the Human Brain Atlas of the Allen Brain Science Institute (21) and other sources (22).

### Experimental data from an animal model of TBI and HS

Examination of gene expression data from a swine model of TBI and Hemorrhagic Shock exhibited functional recovery following VPA therapy showed remarkable enrichment of transcription factors (TFs) that were regulated in a significant manner by the drug. In the animal model, swine were subjected to controlled TBI and hemorrhage (40% blood volume), kept in shock for 2 h, and resuscitated with vehicle or vehicle + VPA (*n* = 3 per group). The vehicle was Hextend (Hospira Inc. Lake Forest, IL) at 50 mL/min *versus* (2) Hextend at 50 mL/min plus VPA at 300 mg/kg (EMD Biosciences Inc., La Jolla, CA). In the Hextend + VPA group, VPA treatment was started 1 h after hemorrhage at an infusion rate of 100 mg/kg per hour intravenously. The vehicle and treatment groups received identical volumes of fluid, which matched the volume of shed blood. After 6 h, brain RNA was isolated, and gene expression profiles were measured using a Porcine Gene ST 1.1 microarray (Affymetrix, Santa Clara, CA).

### Gene expression data from postmortem human brain

Results of microarray expression analysis from postmortem human brain were obtained from different sources (21,22). In a patient cohort with epilepsy, patients on VPA therapy were compared to controls at time of death (TOD) for relative levels of several TFs, nuclear receptors, and other mRNAs (23). Sample sizes for each group were *N* = 6 for epileptic patients on VPA therapy at TOD, *N* = 6 for epileptic patients on a different anti-convulsant drug at TOD.

### Experimental data from the human SK-N-SH cell line

Publicly available date from SK-N-SH cells were used for two applications: (1) For determination of DNAse I hypersensivity and allele bias, as they are included as examples in the deltaSVM machine learning algorithm (19), and (2) Evaluation of spatial interactions in the human genome using data from a high resolution Hi-C dataset (19). To discover how the VPA pharmacogenomic SNPs we selected produced robust pharmacogenomic stratification, evaluation of the chromatin interactions of regulatory elements they were located in was used to preliminarily map the putative VPA pharmacodynamic pathway in human brain.

SK-N-SH cells are a human neuroblastoma cell line which > contains a mixture of at least 2 cell types.

### Published literature on pharmacogenomic associations and VPA targets and pathways

Two hundred and fifty-four peer-reviewed published articles were retrieved between January 1 and October 1 2016, including gene association studies, as well as basic and clinical pharmacology reports. An automated Boolean search string was used in PubMed (24), Google Scholar (23) and clinicaltrials.gov (25), consisting of “valproate OR valproic acid OR sodium valproate OR divalproex sodium AND pharmacodynamics OR mechanism of action OR brain OR pathways AND pharmacogenomic AND bipolar disorder AND epilepsy AND migraine headache AND gene OR SNP AND association AND human.” There are no published genomewide association studies (GWAS) that are focused on medication response or adverse events related to only valproic acid therapy in epilepsy, bipolar disorder or migraine. There are however, several GWAS that have investigated SNPs associated with human populations that have treatment-resistant or refractory epilepsy. These studies are made up primarily of patients who do not respond to a combination of anticonvulsant drugs or worsen on therapeutic regimens. Due to confounding related to polypharmacy these studies were excluded. For this study, we avoided anything but primary research studies in which we re-tested all the parameters and statistical tests that were employed. This dramatically reduced the number of studies from 254 to 52 that could be included in this analysis.

### Analysis

#### VPA pharmacogenomic SNPs

To better understand human CNS pathways that are involved in the mechanism of action of valproic acid, we combined SNP imputation with bioinformatics analysis as performed in a previous study (22). SNPs included in the analysis were those likely to be associated with known biological targets of VPA, could be functionally annotated, and were derived from primary research studies. For this analysis of potential pharmacodynamic pathways, we excluded pharmacokinetic genes and their variants such as the *UGT* and *CYP* super-families. To select VPA pharmacogenomic SNPs we combined SNP imputation from VPA pharmacogenomic association studies, and performed computational and bioinformatics analysis as described in detail (22). DrugBank (25) and the pharmacogenomic mutation database (PGMD®; Qiagen) lists pharmacodynamic targets that have been associated with VPA (26), and these were also interrogated to gather additional information.

In Fig. 1.1, bioinformatics analysis was used to identify the most probable causal SNP from the association regardless of the lead SNP(s) that were reported, and included the following bioinformatics analysis to determine the functionality of the variant (22): (1) Location in open chromatin as indicated by peaks of DNAse I hypersensitivity, (2) Low to moderate methylation of any cytosine residues, (3) The presence of histone marks that indicate regulatory function (H3K27ac + H3K4me1 = enhancer; H3K27ac + H3K4me3 = promoter), (3) The location of the variant within the context of the gene or in an intergenic domain, (4) Whether the regulatory element has yet to be annotated as a molecular quantitative trait locus (eQTL, hQTL, etcetera), (4) Proteins, including transcription factors, bound to the regulatory element as determined by ChIP-Seq indicating its regulatory function, (5) Disruption by the pharmacogenomic SNP of transcription factor binding sites as indicated by alterations in the position weight matrix, (6) Association of the regulatory element with the requisite RNA species (e.g., bi-directional enhancer RNA = enhancer; mRNA = promoter), (7) Connectivity of the regulatory element with other elements in the genome as indicated by the Hi-C chromatin conformation capture method limited to SK-N-SH cells, which are the ENCODE Tier 2.5 neural surrogate cell line, (8) Transcriptional programming by factors which are responsible for determination of neuronal cell fate, (9) Determination of the allele bias of the allelic variant using the deltaSVM algorithm (19), (10) examination of the neuroanatomical distribution of target gene expression data in postmortem human brain using both microarray and *in situ* hybridization data from the Human Brain Atlas of the Allen Brain Science Institute (21).

#### Harmonization of nomenclature: pig, human and rodent genes and their regulatory elements

This study involves cross-species comparisons from pig (*Sus scrofa*), rat (*Rattus norvegicus*) and human (*Homo sapiens*). To ensure that we could reliably use inter-species nomenclature in the context of the human CNS, we compared gene, promoter and enhancer data using 3 resources: (1) comparative assessment of pig, rodent and human transcriptomes for identity (27), (2) thorough analysis of human accelerated regions in human brain that are not present in other mammalian brains for exclusion (28), (3) cross-species assessment of histone modifications, open chromatin and deep learning methods applied to pigs, mice, rats and humans to ensure concordance of identifiable enhancers, promoters, super-enhancers and master TFs (29), and (4) enhancer-promoter loop conservation across species using context‐dependent conservation (30). All gene symbols and homologous regulatory elements were found to be the same, at least in regards to nomenclature, chromatin state annotation, and relative position within the genome. We then harmonized our nomenclature with the results of the roadmap epigenome mapping consortium. Thus, gene and protein symbols and definitions were consistent with HGNC nomenclature, except as indicated by citation in the text. Gene symbols are from GENCODE except where indicated, and genomic location coordinates are from build 38 of the Human Genome.

For annotation, we focused on active enhancers, promoters and transcription start sites, so other chromatin states were not used in the present study, including flanking and poised regulatory elements, transcribed domains or repressed elements. Although potential regulatory variants may be repressed in one tissue or brain region but not another, we could directly assess function in any biological system in this study, so we relied on the data provided by the association studies.

#### VPA-regulated transcription factors (TFs)

Next, we examined data on TFs, including master TFs as defined by super-enhancer regulation in human brain that are regulated by VPA, and TFs and genes that are targets of these Master TFs (see Table I for the complete list of data sources). We examined previously characterized transcriptional networks that contained VPA-responsive TFs, results on conserved network topology from model systems, organisms and mechanisms of neurogenesis (34,35) and known gene regulatory network dynamics (36–38). We also looked at the overlap of VPA genes which encode TFs and HOT (high-occupancy target) regions, which are bound by large number of transcription factors (39). Other data sources are detailed in Supplementary Table 1.

**Table I:** Selected Pharmacogenomic Variants Selected for Allelic Variation in VPA Dose, Response and Pharmacodynamics in Human Populations. Noncoding SNPs that Identified Regulatory Elements, Including Enhancers and Promoters

SNP	GENE^a^	TYPE	deltaSVM SCORE^b^	DISEASE	EFFECT	EFFECT SIZE	REF
rs2857654_A	*CCL2*	Enhancer	−2.275704	Epilepsy	Response in children	1.45 (1.06–1.99)	(31)
rs3764028_G	*GRIN2B*	Promoter	−5.097029	Epilepsy	Dose range	1.7553 (1.219–2.291)	(32)
rs2269577_G	*XBP1*	Promoter	4.710044	BPD	Response^c^	1.2754 (0.329–2.221)	(33)

^a^RefSeq nomenclature

^b^deltaSVM is a machine learning algorithm that determines the causal nature of gene variants, including DNAse I hypersensitivity and allele bias (21)

^c^Total treatment response score, Kruskal–Wallis test for valproate prophylactic treatment response. BPD: Bipolar disorder, sample containing patients with BPD 1 and BPD 2. Effect sizes generated using Cohen’s D-test adjusted for sample size

#### Chromatin remodeling proteins and complexes

Several and chromatin remodelers, including *ARID1A, BCL11A, CHAF1B,* were among genes regulated by VPA. We determined the regulatory targets of these genomewide, and determined and to what degree they overlapped the VPA-responsive promoters as part of the reconstruction of the network. We also examined pioneer factors such as PAX6, which appears to act in concert with chromatin remodeling complexes during neurogenesis, including published data on TFs that are pioneer factors and chromatin state regulators (Supplementary Table 1).

#### Reconstruction of VPA gene regulatory networks in the human CNS

Figure 1.2 shows the method by which we reconstructed the central VPA pathway in the human CNS. It involved: Identification of databases relevant to modeling the VPA regulatory circuit, Data aggregation from public databases and in-house data, manual curation of the data in the context of a VPA central mechanism of action, preparation of curated data for model integration, iterative gene set enrichment analysis (GSEA) using different public and commercial software, and pathway analysis using IPA® and the STRING database of protein-protein interactions (40), as well as network analysis using weighted gene co-expression network analysis (WGCNA) in R (41) and node-edge modeling systems modeling using Python (42). In addition to applying redundant software analysis tools, we also used different open source and commercial databases, including IPA® (43), Pathway Commons (44) and Reactome (45) databases as well as manual curation of the scientific literature, to determine network interactions.

For reconstruction of VPA’s gene CNS regulatory pathway, we used a hybrid model development method that combines GSEA and pathway analysis/network modeling software used in bioinformatics with a technique for constructing core regulatory circuitry which includes super-enhancers, TFs and auto-regulation, based on defined biological attributes of transcriptional regulation in the human CNS (Supplementary Table 1). During the project, we discovered several other TFs that are regulated by VPA that directs cell fate, in addition to many genes whose was expression was regulated by ASCL1 (Achaete-Scute Family BHLH Transcription Factor 1), NEUROD1 (Neuronal Differentiation 1), MEF2C (Myocyte Enhancer Factor 2C), MEF2D (Myocyte Enhancer Factor 2D), MYT1L (Myelin Transcription Factor 1 Like), and TBR1 (T-Box, Brain 1). Many TFs were tightly coupled in terms of target promoters, many of which were also involved in neurogenesis and neuroplasticity. As such, this convoluted super-program was investigated in greater detailed.

#### Relationship between VPA-regulated TFs and other TFs in human brain and cell types

To understand the relationship between genes whose expression is highly regulated by VPA following brain injury in the CNS, we first examined sets of TFs which were known to be differentially regulated by VPA in brain and whose function was linked to neurogenesis and suppression of gliogenesis in human brain. Comparisons were made between VPA-responsive master TFs and their targets and other TFs in the human telencephalon, and the different sets are represented on a coronal section of the human telencephalon (Fig. 4). Adult human brain regions, examined in the roadmap epigenome mapping consortium (15), included the angular gyrus, anterior caudate, cingulate gyrus, hippocampal formation, inferior temporal lobe and mid-frontal lobe. Cell types included astrocytes and HUVEC, the latter a lymphoblastoid cell line derived from human vascular endothelial cells.

TFs were assigned using a subset of ENCODE brain regions and in astrocytes based on annotation of super-enhancer status using histone marks such as H3K27ac and other characteristics (15,17). From this analysis, only 8 of the TFs and nuclear receptors regulated by VPA exhibit enhancer-like attributes in various brain regions. Other enriched TFs include NFIX, regulated by CHAF1B and SOX2. The correlation between VPA-TFs and TFs which function with superenhancer status in different human brain regions is obvious, with this small subset accounting for 31–48% of all TFs across the telencephalon. In astrocytes, as shown in Fig. 4, although only MEF2D is represented as a VPA-TF, 17% of all TFs in this cell type are regulated by VPA-TFs. HUVEC cells were used as a surrogate for CNS endothelial cells, and MEF2C, MEF2D and NR1D1 are VPA-TFs, with these TFs and their targets accounting for 18% of the TFs in this lymphoblastoid cell line

## RESULTS

### VPA regulation of gene expression following injury to the brain in a swine model

Most the genes differentially-regulated by VPA were several master TFs that are highly expressed during development of the CNS, and are also found in the developing adult human telencephalon. TFs that target the greatest number of differentially expressed genes were NEUROD1, ASCL1, MEF2C, MEF2D, BCL2L11 (BCL2 Like 11), ELK1 (ELK1, ETS Transcription Factor), MYT1L, PPARD (Peroxisome Proliferator Activated Receptor Delta), SIRT1 (Sirtuin 1), SMARCB1 (SWI/SNF Related, Matrix Associated, Actin Dependent Regulator Of Chromatin, Subfamily B, Member 1), and TF (Transferrin). All of these genes except for *SMARCB1,* which is induced by VPA, were found in the VPA-regulated set.

**Fig. 2:**
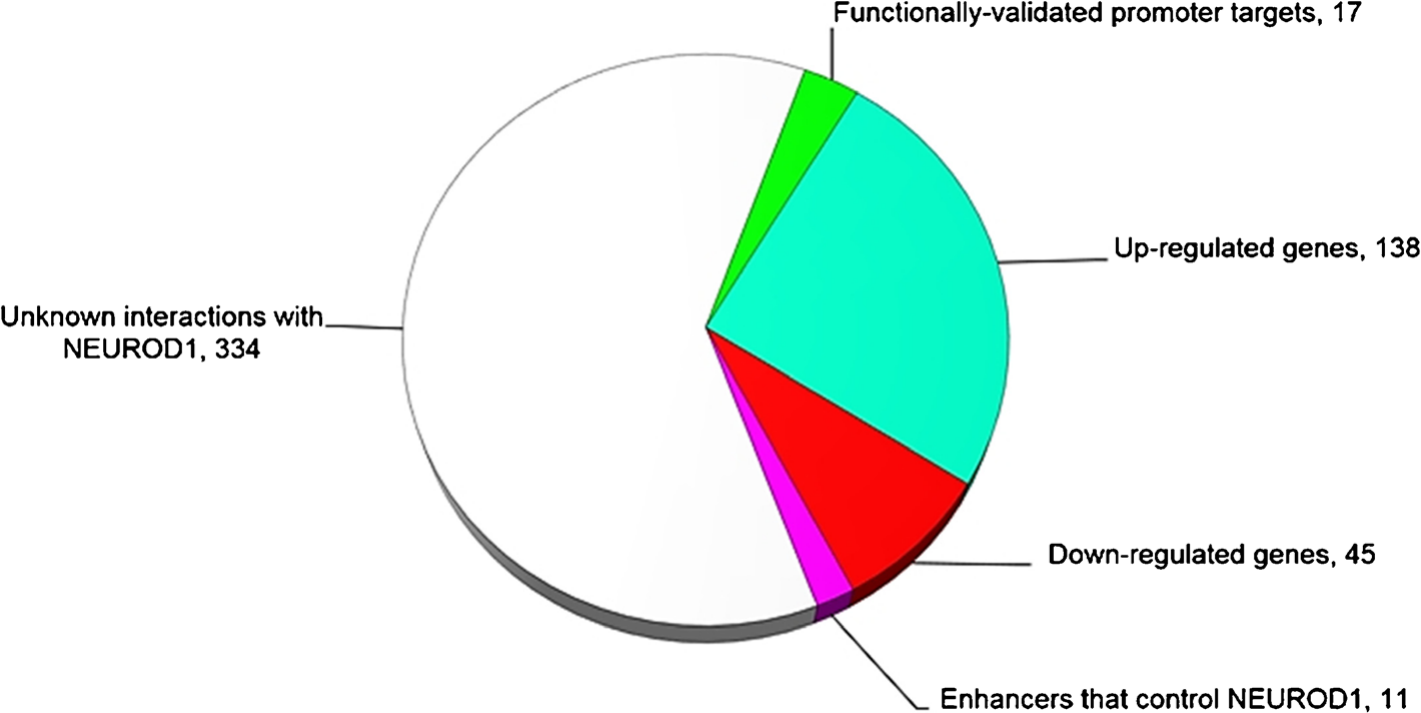
Types of NEUROD1 control of genes differentially regulated by VPA in adult swine brain following injury. The 11 enhancers that control NEUROD1 include genes that are co-regulated by the same enhancer as NEUROD1 and genes which harbor single gene variants that impact enhancers that affect NEUROD1 gene expression. For details see Supplemental dataset.

Analysis of significantly up- or –down-regulated genes in the porcine model of TBI and HS showed that NEUROD1 was involved in the regulation of 63% of the regulated genes in this dataset. Using Fisher’s exact test, the *p*-value of *NEURDO1*-regulated genes in the dataset *versus* genes with those not known to be regulated by *NEUROD1* is *p* = 1.6E-05. Figure 2 shows the distribution of non-overlapping NEUROD1 interactions in this dataset. The most common target of overlapping TFs was *CASP3* (Caspase 3), which is the target of 8 different TFs, and *SIRT1*, *PPARD* and *TBR1*, all targeted by 5 different TFs. Common target TFs and nuclear receptors involved in development and neuronal programming included the up-regulated genes *NR6A1* (Nuclear Receptor Subfamily 6 Group A Member 1; 1.75 log^2^-fold), *BCL11A* (B-Cell CLL/Lymphoma 11A; 1.6 log^2^-fold), *MYT1L* (Myelin Transcription Factor 1 Like; 1.6 log^2^-fold), *TBR1* (1.49 log^2^-fold), *NR1D1* (Nuclear Receptor Subfamily 1 Group D Member 1, 1.42 log^2^-fold) and *MEF2C* (1.42 log^2^-fold). Among VPA-responsive genes that were up-regulated, there was also significant representation of thyroxine-responsive genes, neurotransmitter genes including corticotrophin releasing hormone, and genes which encode proteins involved in neuronal cell adhesion, synaptogenesis, axonal growth, dendritic arborization and the cytoskeleton. For additional information about programmers of cell fate regulated by VPA following CNS injury, please refer to the Supplement.

TFs that program oligodendrocytic cell fate, endothelial cell fate and angiogenesis were significantly down-regulated compared to other TFs in this dataset (*p* = 2.78E-04; Fisher’s exact test). These included *FGF7* (Fibroblast Growth Factor 7; −3.2 log^2^-fold), PROX*1* (Prospero Homeobox 1; −1.81 log^2^-fold), *SOX10* (SRY-Box 10; −1.81 log^2^-fold), *PRRX1* (Paired Related Homeobox 1; *BMP2* (Bone Morphogenetic Protein 2; −1.73 log^2^-fold) and *ST18* (Suppression Of Tumorigenicity 18, Zinc Finger; −1.22 log^2^-fold). Other significantly down-regulated genes included *CYR61* (Cysteine Rich Angiogenic Inducer), *OPALIN* (Oligodendrocytic Myelin Paranodal And Inner Loop Protein), PMP*2* (Peripheral Myelin Protein 2) and *THBS1* (Thrombospondin 1).

Supplementary Figure 3 shows the significant functions of the 50 most up-regulated and most down-regulated genes following VPA therapy in a swine model of TBI and hemorrhage. These results show that in an animal model of TBI and hemorrhage, VPA therapy induces genes associated with neurogenesis and neuroplasticity, and represses those associated with cell loss, endothelial cell invasion, and angiogenesis.

### Selection of VPA pharmacogenomic SNPs for further examination

Table I shows VPA pharmacogenomic SNPs that were selected for further analysis. These SNPs stratify response to VPA in human populations, and all 3 are located in regulatory domains (promoters or enhancers), and exhibit significant chromatin allele bias as measured by the deltaSVM algorithm (19). They include:

1. The intronic SNP rs2857654_A located within the *CCL2* gene, which encodes the Chemokine C-C Motif Ligand 2, and is most significantly associated with cell movement and migration of cells and this enhancer interacts with VPA (31);
2. The 5′ SNP rs3764028_G located in the distal *GRIN2B* promoter (32). Spatial mapping in SK-N-SH cells shows that it forms an inter-chromosomal transcriptional hub (Fig. 3a), contacting genes in *cis*- and *trans*- in SK-N-SH cells that are enriched for genes involved in neuronal differentiation and development of the CNS (Fig. 3a);

**Fig. 3:**
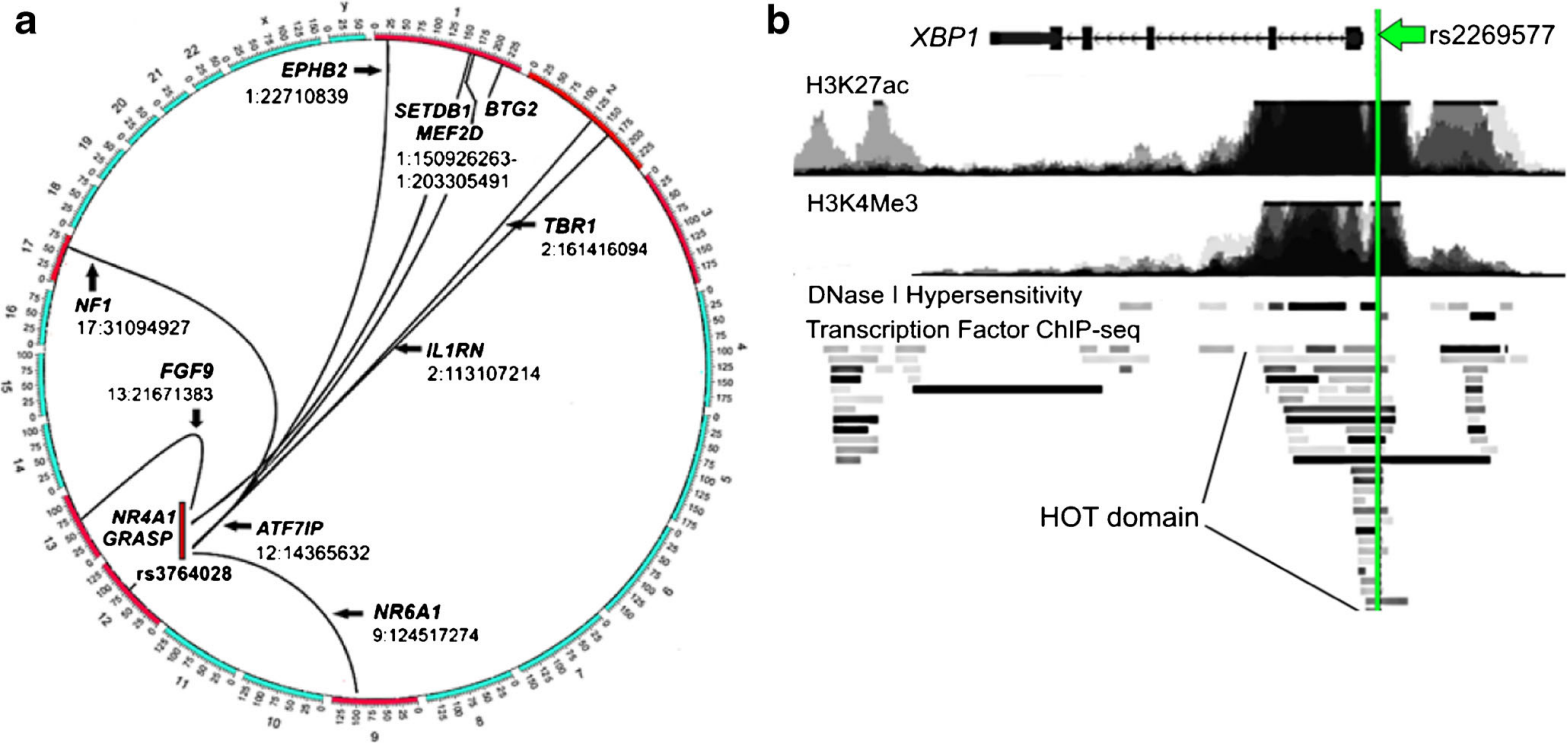
Spatial context of promoters containing VPA pharmacogenomic SNPs. **(a)** Whole genome plot of transcriptional hub based on *cis*- and *trans*-contacts of the SNP rs3764028_G in the promoter of the *GRIN2B* gene based on data from Hi-C mapping of this SNP in the human neuronal cell line SK-N-SH. **(b)** Relative location of the VPA pharmacogenomic SNP rs2269577 (*green line*), with histone marks H3K27ac and H3K4me4 indicative of an active promoter, location in a DNase I hypersensitivity region indicative of open chromatin, and overlapping a HOT domain containing many TFs (39). This SNP is located within a TAD located on chromosome 22 (26,600,000–28,000,000) (46) containing the *XBP1* gene as well as other genes. Inset. Screenshots from the UCSC genome browser, build hg18, taken from the UCSC genome browser in **(b)** (47).
3. The 5′ SNP rs2269577_G located in the promoter of the *XBP1* (X-Box Binding Protein 1) gene (33), which functions as a transcription factor during endoplasmic reticulum (ER) stress by regulating the unfolded protein response. Required for cardiac myogenesis and hepatogenesis during embryonic development, and the development of secretory tissues such as exocrine pancreas and salivary gland. An enhancer co-regulates *XBP1*, *EWSR1* (EWS RNA Binding Protein 1), *CCDCC117* (Coiled-Coil Domain Containing 117), *KREMEN1* (Kringle Containing Transmembrane Protein 1) and *ZNRF3* (Zinc And Ring Finger 3) genes, which are co-localized within a TAD located on chromosome 22 (46). The location of this SNP 5′ to the *XBP1* gene is shown in Fig. 3b.

### VPA modulates a transcriptional hub consisting of 12 genes in spatial contact with the *GRIN2B* promoter

The 5′ SNP rs3764028_G located in the distal *GRIN2B* promoter is in a region associated with massive chromatin reorganization in brain (48,49) in rodents and human cell lines. This SNP detects a promoter that maintains spatial contact, as determined by Hi-C maps of SK-N-SH cells. SK-N-SH cells are a lymphoblastoid cell line which contains a mixture of 2 cell types. This regulatory community also contains 3 known enhancers including 2 super enhancers as well as a promoter with spatial contacts to genes associated with neuronal survival and plasticity. This replicates a result from a study in mouse brain in which researchers combined genome-wide analysis of data sets for chromatin accessibility (FAIRE-Seq) and the enhancer mark H3K27ac, in which they found a subset of genes associated with neuroprotection and plasticity that increased transcription in adult mouse brain following activation of the glutamate receptor (49). In our study, both gene set enrichment using Gene Ontology, as well as manual inspection of genes that maintain spatial contacts with the promoters and enhancers detected by our putative causal SNPs demonstrate selectivity for neuroplasticity and chromatin reorganization. In agreement with their findings, genes whose abundance increased included *NR4A1*, which regulates dendritic spindle density and organization in human brain following neuronal excitation, *BTG2*, a transcription factor that inhibits neural precursor cell proliferation and stimulates neuron cell differentiation and acts in histone arginine methylation, *ILRN*, which is also in contact with an enhancer associated with SNP rs2857654_A, and *NF1*, which differentially controls neural stem cell proliferation. In addition, we identified contacts with *GRASP*, part of a receptor complex scaffold that regulates G protein-coupled glutamate receptor signaling, *MEF2D*, which is regulated by NEUROD1, and *EPHB2* (EPH Receptor B2), a developmentally-regulated receptor tyrosine kinase that functions in axonal guidance during development. This promoter also maintains long distance spatial contact with *SETDB1*, an H3-K9 histone methyltransferase that regulates epigenetic gene silencing to maintain stem cell pluripotency, a result that has been experimentally shown in rodent brain and cell lines by other researchers (48,49).

The distal *GRIN2B* promoter maintains spatial interactions with genes that program neuronal cell fate in humans, and are highly responsive to VPA. These are *TBR1* (T-Box, Brain 1), which is up-regulated by VPA following TBI in our animal model. TBR1 is a TF that acts as a potent programmer of neurogenesis (50), controls the differentiation of pyramidal cells in neocortex, and controls expression of the *GRIN2B* gene in developing cerebral cortex (51). The other is *FGF9* (Fibroblast Growth Factor 9), which is produced by developing neurons to maintain homeostasis within the surrounding milieu. It plays an important role in the regulation of embryonic development, cell proliferation, cell differentiation and cell migration, regulation of gliosis during repair and regeneration of brain tissue after damage and the differentiation and survival of neuronal cells (52). Several of these genes that exhibit spatial proximity to the *GRIN2B* promoter in human SK-N-SH cells are also up-regulated by VPA in the swine model of TBI and hemorrhage. These include *FGF9, NR6A1, TBR1* and *MEF2D*.

### Relationship between VPA-regulated TFs and other TFs in human brain and cell types

To understand the relationship between genes whose expression is highly regulated by VPA following brain injury in the CNS, we first examined sets of TFs which were known to be differentially regulated by VPA in brain and whose function was linked to neurogenesis and suppression of gliogenesis in human brain. Comparisons were made between VPA-responsive master TFs and their targets and other TFs in the human telencephalon, and the different sets are represented on a coronal section of the human telencephalon (Fig. 6). Adult human brain regions included the angular gyrus, anterior caudate, cingulate gyrus, hippocampal formation, inferior temporal lobe and mid-frontal lobe. Cell types included astrocytes and HUVEC, the latter a lymphoblastoid cell line derived from human vascular endothelial cells.

**Fig. 4:**
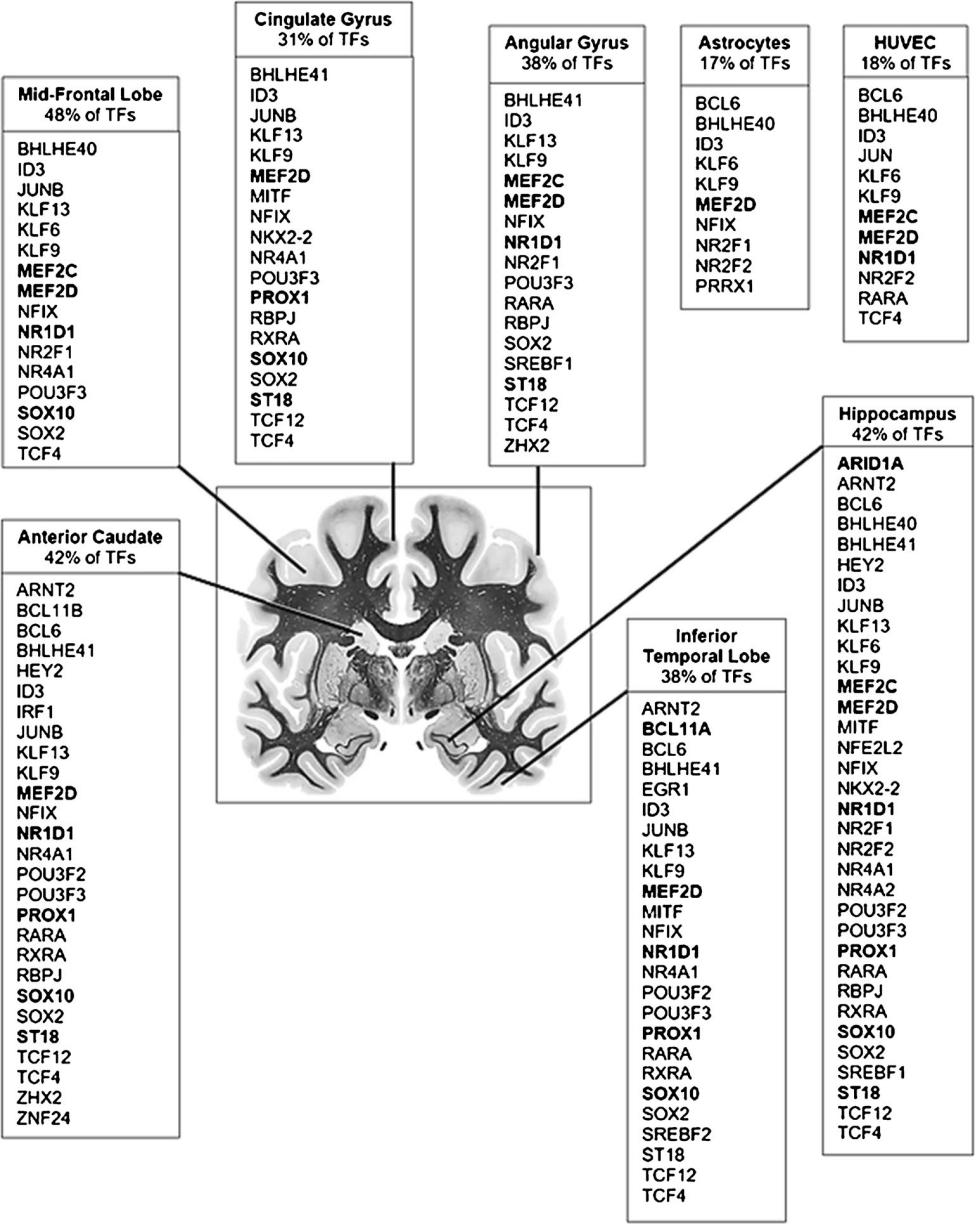
Distribution of VPA-regulated master TFs and their target TFs in the VPA pathway mapped onto a coronal section of human brain. TFs in *bold* are VPA-responsive master TFs, which are regulated by super-enhancers according to H3K27ac marks and other attributes, based on Supplementary Table 3 and (53). Percentages indicate total number of VPA-regulated TFs that have been annotated as such in each region.

TFs were assigned using a subset of ENCODE brain regions and in astrocytes based on annotation of super-enhancer status using histone marks such as H3K27ac and other characteristics. From this analysis, only 8 of the TFs and nuclear receptors regulated by VPA exhibit enhancer-like attributes in various brain regions. Other enriched TFs include NFIX, regulated by CHAF1B and SOX2. The correlation between VPA-TFs and TFs which function with superenhancer status in different human brain regions is significant (*p* = 3.12E-15), with this small subset accounting for 31–48% of all TFs across the telencephalon. In astrocytes, as shown in Fig. 4, although only MEF2D is represented as a VPA-TF, 17% of all TFs expressed in this cell type are regulated by VPA-TFs. HUVEC cells were used as a surrogate for CNS endothelial cells, and MEF2C, MEF2D and NR1D1 are VPA-TFs, with these TFs and their targets accounting for 18% of the TFs in this lymphoblastoid cell line.

Examination of the interaction and correlation between diverse datasets led to a definitive set of master TFs and their target TFs that were involved in VPA-mediated neurogenesis. Forty-seven TF targets of the 18 master transcription factors regulated by VPA include are controlled by 18 master TFs (Supplementary Table 2), but not all the master TFs are expressed in the adult human telencephalon at high abundance (Fig. 4). These data show that VPA-regulated TFs responsible for neuronal cell fate are found in the adult human brain, and TFs that can program fibroblasts into oligodendrocytes including OLIG2 and ST18, are also present. For details concerning Fig. 4 see (53).

Next, we performed a GSEA on VPA-responsive *versus* non-VPA TFs found in these different adult brain regions and compared these results to those found in our animal model. Supplementary Figure 4 shows a comparison of gene set enrichment, including brain region subsets from the data used for Fig. 4, including (Supplementary Figure 4A), the set of VPA-regulated TFs and (Supplementary Figure 4B) the set of non VPA-responsive TFs. TFs found in human telencephalon that are not VPA-responsive are not enriched for neurogenesis, but are enriched for differentiation and development of other cell types.

### Preliminary evidence from microarray expression data from postmortem human brain tissue

**Fig. 5:**
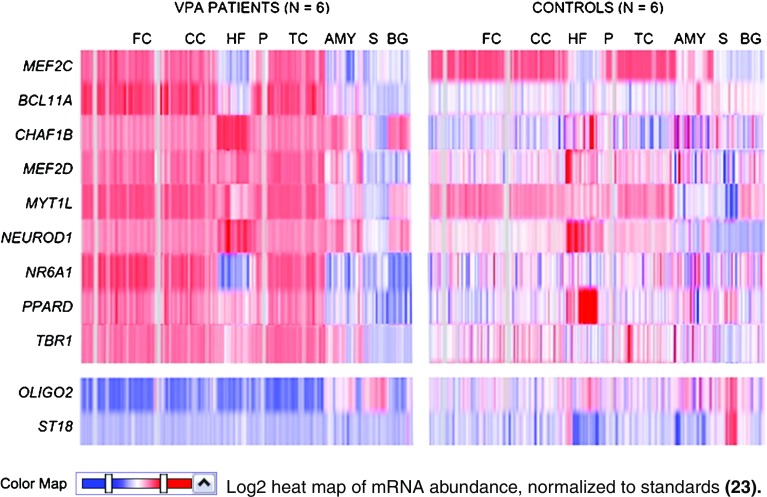
Differential regulation of TFs, nuclear receptor and chromatin remodeler involved in specification of cell fate in patients on VPA therapy at TOD *versus* controls who had no recorded history of psychotropic medication at TOD. These data obtained from a study of patients with epilepsy, and medication-free control individuals, showing samples from different regions of the telencephalon. Vertical stripes in each brain region indicates data from individual patients. AMY: Amygdala; BG: Basal ganglia; CC: Cingulate cortex; HF: Hippocampal formation; FC: Frontal cortex; P: Parahippocampal gyrus; TC: Temporal cortex; S: Septum. Courtesy, Allen Brain Science Institute (21).

As part of a larger study examining patients with epilepsy, we obtained limited data on TF gene expression on 6 patients who were on VPA therapy at time of death (TOD) from the Human Brain Atlas of the Allen Brain Science Institute (23). These were compared to data from individuals who had no known medication history. Figure 5 shows a comparison between individuals on VPA treatment at TOD *versus* controls. As can be seen from this heatmap, patients on VPA had elevated levels of TFs that are responsible for neuronal cell fate specification, but reduced *OLIG2 and ST18* gene expression, which are involved in the specification of glial cell fate during CNS development. Although *MEF2C* is involved in neurogenesis and patterning of cortical structure, the heatmap shows its expression appears to be slightly less in VPA patients than in controls, especially in the amygdala. In contrast, the abundance of *BCL11A, CHAF1B, MEF2D, MYT1L, NEUROD1, NR6A1, PPARD* and *TBR1* mRNA was significantly higher in these brain regions than in epileptic patients who were taking VPA at TOD than in control individuals (*p* = 4.2E-07).

### Sub-network analysis

**Fig. 6:**
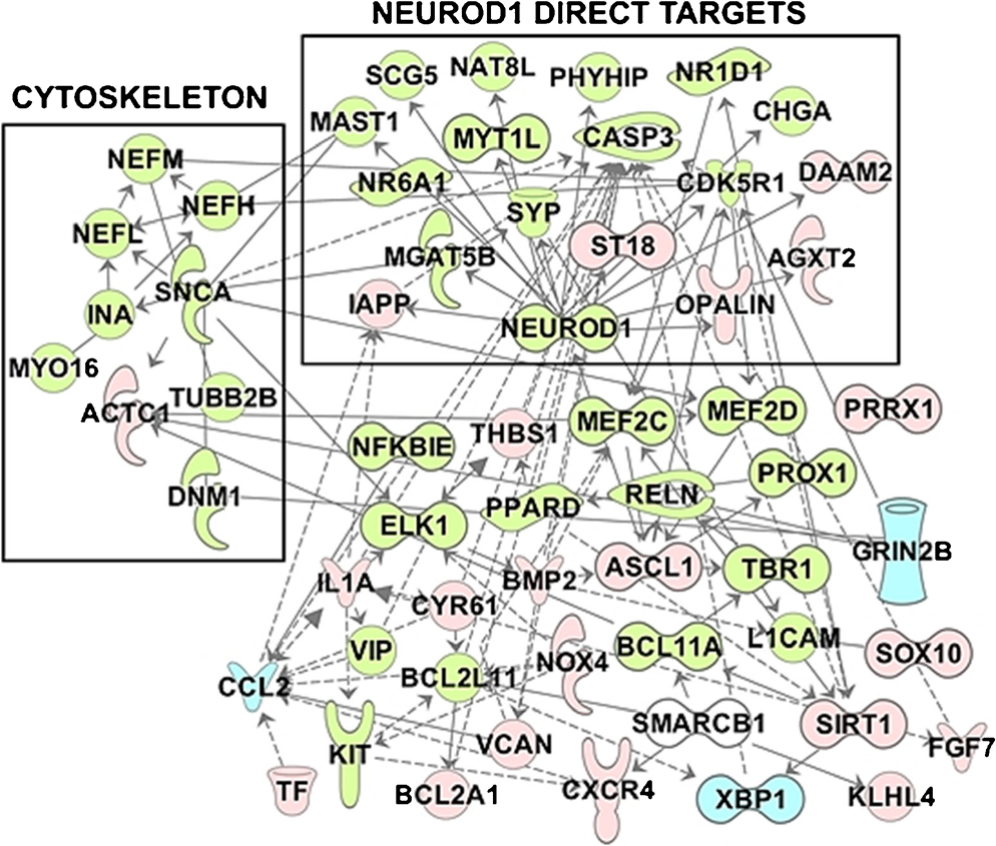
Examples of subnetworks in network reconstructed from injured swine brain treated with VPA. These include 11% of the differentially-regulated molecules in the dataset, enriched for TFs, nuclear receptors and VPA pharmacogenomic genes (*blue*). Insets include a fraction of the genes associated with the cytoskeleton and nucleoplasm, and the 17 functionally-validated promoter targets of NEUROD1. Symbols in *green* indicate genes up-regulated by VPA in this model and the *red* indicate down-regulated genes. For extension of this network to the VPA pharmacodynamic pathway in human brain, see Supplementary Figure 1. In the entire dataset, the genes that discretize VPA response include *CCL2*, with 23 direct interactions, *GRIN2B*, with 20 direct interactions, and *XBP1* with 4 direct interactions. Pathway contacts made using the “connect” function of IPA® (40). For symbol key, see Supplementary Figure 1.

Analysis of VPA gene expression from expression data from the injured swine brain was made using several approaches, and several important sub-networks are shown in Fig. 6. Subsequently reconstruction of a network of the central VPA pathway in human brain that extended the animal model included genes that encode known TFs that direct cell fate in the developing CNS in the human, a subset of nuclear receptors, cytoskeletal proteins including NEFL, NEFH and INA, circadian genes, genes whose products are associated with histone acetylation, suppression of HDACs, apoptotic proteins, neurotransmitters including 15 potassium channels, corticotropin releasing hormone, cholinergic receptors, GABA receptors, opiate receptors, synaptic proteins, proteins that promoter neurite extension, axonal growth and dendritic arborization, anti-angiogenic proteins, growth factors and others. Supplementary Figure 1 shows the reconstructed VPA gene regulatory network of TFs in human brain as determined by IPA® including master TFs, TFs, nuclear receptors, growth factors, selected VPA pharmacogenomic genes and related genes that are up- or down-regulated following TBA in an animal model, as well as additional genes determined by IPA® that are intimately associated with these genes in human brain using the “grow” function of the software. This forms the transcriptional foundation of the central VPA pathway in human brain. Although between 28 and 50% of VPA-inducible master TFs are normally expressed in adult brain (Fig. 6) there are TF genes that are not normally expressed at high abundance that are responsible for activation of neurogenesis and suppression of cell fate specification of non-neuronal cell types that are up-regulated following VPA therapy. The STRING database (40) leads to the same or a very similar network of TFs, as shown in Supplementary Figure 2. Following reconstruction of the network, we examined which drugs were most likely to impact this pathway based on existing data. Analysis using the IPA® “upstream regulatory” toxicology analysis, the most significant drug/chemical regulating this network was VPA (*p*-value 2.98E-22 using Fisher’s exact test).

Supplementary Figure 1 only includes a fraction of the pathway, including direct targets such as master TFs and some of the genes they regulate, and represents a union of data from human and a swine model of TBI and hemorrhage. Green symbols show up-regulated genes following VPA treatment *versus* vehicle in the swine model of TBA, and red symbols are down-regulated genes following VPA treatment in the animal model of brain injury. Blue symbols are VPA pharmacogenomic genes (Table I). Symbols lacking colors are VPA-regulated genes that were predicted by IPA® using the “connect” function, as essential components of the same interconnected pathway expressed in human brain. We then compared overlap of pathway components to previously defined networks in the Reactome database (45), the IPA® database (43) and Gene Ontology (54). Previously defined biological networks across tissues and species exhibited significant overlap with the set of VPA pathway molecules. As might be expected from the widespread action of VPA-responsive TFs that direct neurogenesis, the 3 most significant overlaps were: (1) Neuronal cell fate determination in mammals (*p*-value < 1.30E-13; *q*-value < 4.39E-10), (2) Nuclear receptor transcription pathway in humans (*p* < 3.94E-13; *q* < 1.67E-10) and (3) Neurogenesis in humans (*p* < 5.17E-12; *q* < 7.18E-10). Supplementary Table 2 shows a summary of the overlap of TFs found in this reconstructed VPA gene regulatory network with a matrix of known TFs responsible for: (1) CNS developmental processes, (2) Specification of germinal layers of cerebral cortex, and (3) Reprogramming/trans differentiation of different cell types to neuroblasts, neural crest, neural progenitor cells and neurotransmitter-specific neurons.

### Many TFs in the VPA regulatory network are located in HOT regions

It has been found that many master TFs regulated by super-enhancers are also sites of high TF occupancy (HOT regions) (39). HOT regions correlate with decreased nucleosome density and increased nucleosome turnover and are primarily associated with open chromatin. Collectively, these HOT regions span approximately 19% of the genome in other species (39,55). Since the reconstructed VPA gene regulatory pathway contains not only master TFs normally found in adult brain (Fig. 4), and the drug induces numerous master TFs in human brain that are expressed at high abundance during CNS development, we examined the overlap between HOT regions and genes in our pathway. Many genes in the reconstructed VPA pathway were in HOT regions.

## DISCUSSION

The rapid action of VPA in the adult brain appears to reduce the impact of ensuing trauma following TBI and hemorrhage, and mitigates neuronal atrophy in bipolar disorder and epilepsy. This raises the question of whether newly differentiated neurons are absorbed into active neuronal regulatory networks to become part of functional neurophysiological pathways. The highly significant changes related to neuroplasticity that is observed in our animal model of TBI and hemorrhage suggest this may be true. However, it is most probable that VPA acts through different pathways following traumatic injury than that observed in healthy tissue. Previous studies in rodent models of ischemia followed by treatment with HDAC inhibitors including VPA and sodium butyrate have observed activation of neurogenesis in the adult brain (10,56), although repair mechanisms may depend on the post-injury timeframe (56).

**Fig. 7:**
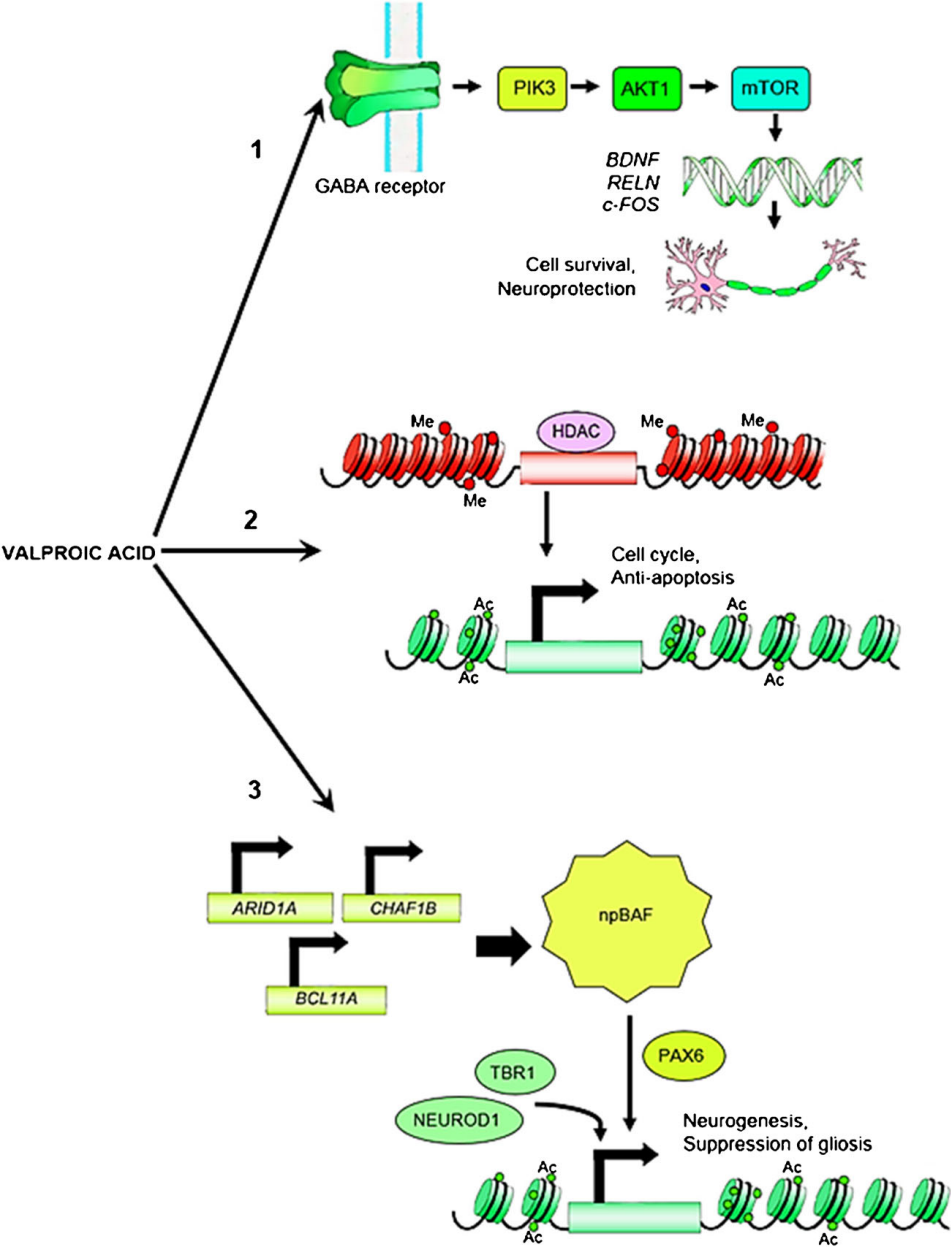
Putative mechanisms of VPA in human brain. **(1)** Activation of AKT1/mTOR signaling pathways by GABA receptors and/or growth factors that activate genes whose expression provides neuroprotection, **(2)** VPA acts directly to open chromatin through HDAC inhibition, leading to histone acetylation and widespread gene expression including expression of genes involved in the cell cycle, and **(3)** Chromatin state remodeling complexes such as npBAF containing proteins encoded by ARID1A, BCL11A and CHAF1B, act as intermediaries to prepare for pioneer TFs to initiate neurogenesis and repression of non-neuronal gene expression. There is evidence that PAX6 acts as a pioneer factor in concert with BAF during neurogenesis (61,62). These mechanisms are not mutually exclusive

Our results show that existing sources of disparate data can be used to model transcriptional networks that are activated by VPA in the human CNS. VPA generates widespread changes in chromatin state in the genome accompanied by targeted transcriptional changes that mediate neurogenesis (12). Although VPA in combination with other small compounds may be able to induce neurogenesis (57), previous research suggests that it acts, in part, through TFs responsible for determination of neuronal cell fate, such as NEUROD1 and TBR1 ((58): Supplementary Table 3). Specific chromatin modifying protein complexes that include ARID1A are critical for chromatin remodeling during neurogenesis (59,60), including acetylation of the histone mark H3K27, which defines active enhancers and promoters. Based on these findings we have devised a model that attempts to explain VPA’s mechanism of action in the human adult brain (Fig. 7). This model consists of 3 components: (1) Activation of AKT1/mTOR signaling pathways by GABA receptors and/or growth factors that activate genes whose expression provides neuroprotection, (2) Opening of chromatin through HDAC inhibition, leading to histone acetylation and widespread gene expression including expression of genes involved in the cell cycle, and (3) Induction of chromatin state remodeling complexes such as npBAF containing proteins encoded by ARID1A, BCL11A and CHAF1B, which act as intermediaries for pioneer TFs to initiate neurogenesis and repression of non-neuronal gene expression. These mechanisms of action are not mutual exclusive and occur concurrently with suppression of TFs such as ST18, which is responsible for glial cell fate programming during development (63).

In cell lines, treatment with VPA causes rapid and extensive chromatin decondensation, suggesting that VPA broadly and directly acetylates histones for transformation of chromatin state from heterochromatin to euchromatin. Thus, it may be that VPA acts directly to inhibit histone deacetylases. Alternatively, rapid activation of chromatin remodeling proteins such as the ARID1A component of the neural progenitors-specific chromatin remodeling complexes npBAF and nBAF may mediate chromatin remodeling following administration of VPA. Ultimately, it may be that all of these mechanisms contribute to VPA’s mechanism of action in the human CNS.

Adequate data now exist to elucidate the transcriptional program that resolves all of the components that are necessary and sufficient to determine VPA’s mechanism in the human brain using knowledge-based discovery. This capability has been demonstrated in other domains, including modeling of the core regulatory circuits in different human tissues and cell lines, including super-enhancers, master TFs and TFs responsible for specifying cell fate (Supplementary Tables 1, 3). In this study, we identified important master TFs such as NEUROD1 and TBR1 that are induced by VPA following TBI in adult brain, but normally expressed at low levels in the mature human telencephalon.

Preliminary data from postmortem human brain must be considered preliminary, as complete medical histories are not available on either patients or controls, there is heterogeneity in the data, and this microarray expression reflects variability associated with a single time point in the analysis of postmortem human brain tissue lacking cause of death in most cases. In addition, although controls were selected based on lack of medication history, medical history was not well characterized.

Since VPA relieves symptoms in disorders such as bipolar disorder, which involves neuronal loss and neural atrophy (64), it is tempting to speculate that VPA may have a positive effect on a range of damaged brain tissue, from mild atrophy to the critical damage that is often observed following TBI. Conversely, VPA may generate aberrant hyperplasia of different cell types in human brain as has been suggested (65). This could account for rebound seizures and CNS impairment including encephalopathy after chronic treatment with VPA (66).

Further investigation is warranted to completely understand how VPA and other HDAC inhibitors can provide functional recovery after severe trauma to the human brain and spinal cord, and to a lesser extent, suppress epileptogenesis, alleviate mania in bipolar disorder, and act as an analgesic in migraine. Modeling using rich data resources as was performed in the current study generate testable hypotheses for more focused experiments in subsequent studies.

## Electronic supplementary material

Below is the link to the electronic supplementary material.

ESM 1 (DOCX 2041 kb)

## Abbreviations

ChIP-seq: ChIP-sequencing is a method used to analyze protein interactions with DNA
eQTL: Expression quantitative trait loci
GWAS: Genomewide association studies
hQTL: Histone quantitative trait locus
H3: Histone 3, one of the 5 main histone proteins involved in the structure of the nucleosome in the chromatin of eukaryotic cells
H3K9me2/3: Histone H3 di- or tri-methylated at residue lysine 9. histone marks that are found in facultatively repressed genes
H3K27ac: Histone H3 acetylated at residue lysine 27. H3K27ac is associated with the higher activation of transcription and therefore defined as an active mark of both enhancers and promoters
H3K27me2/3: Histone H3 di- or tri-methylated at residue lysine 27. histone marks that are found in facultatively repressed genes
H4: Histone 4, one of the 5 main histone proteins involved in the structure of the nucleosome in the chromatin of eukaryotic cells
HDAC: Histone deacetylase
HOT: High occupancy by transcription factors. indicative of enhancers and promoters, especially in developmentally important genes
HS: Hemorrhagic shock
IC_50_: Concentration of an inhibitor where the response (or binding) is reduced by half
TAD: Topologically-associating domain, the basic spatial unit of transcription in the human genome
TBI: Traumatic brain injury
TF: Transcription factor
SK-N-SH: SK human caucasian neuroblastoma, an immortalized human neuronal cell line
VPA: Valproic acid

## Gene symbols

ARID1A: AT-rich interaction domain 1A
ASCL1: Achaete-Scute family BHLH transcription factor 1
ATF7IP: Activating transcription factor 7 interacting protein
BCL11A: B-Cell CLL/Lymphoma 11A
BCL2L11: BCL2 like 11
BMP2: Bone morphogenetic protein 2
BTG2: BTG family member 2
CASP3: Caspase 3
CCDCC117: Coiled-coil domain containing 117
CCL2: Chemokine C-C Motif Ligand 2
CHAF1B: Chromatin assembly factor 1 subunit B
CTCF: CCCTC-binding factor
CYR61: Cysteine rich angiogenic inducer
ELK1: ELK1 ETS transcription factor
EPHB2: EPH receptor B2
EWSR1: EWS RNA binding protein 1
FGF7: Fibroblast growth factor 7
FGF9: Fibroblast growth factor 9
GRASP: GRP1 (General receptor for phosphoinositides 1)-associated scaffold protein
GRIN2B: Glutamate ionotropic receptor NMDA type subunit 2B
IL1RN: Interleukin 1 receptor antagonist
KREMEN1: Kringle Containing Transmembrane Protein 1
MEF2C: Myocyte enhancer factor 2C
MEF2D: Myocyte enhancer factor 2D
MYT1L: Myelin transcription factor 1 Like
NEUROD1: Neuronal differentiation 1
NF1: Neurofibromin 1
NR1D1: Nuclear receptor subfamily 1 group D member 1
NR4A1: Nuclear receptor subfamily 4 group A member 1
NR6A1: Nuclear receptor subfamily 6 group A member 1
OPALIN: Oligodendrocytic Myelin Paranodal and Inner Loop Protein
PAX6: Paired box 6
PMP2: Peripheral Myelin Protein 2
PPARD: Peroxisome proliferator activated receptor delta
PROX1: Prospero homeobox 1
PRRX1: Paired related homeobox 1
SETDB1: SET domain bifurcated 1
SIRT1: Sirtuin 1
SMARCB1: SWI/SNF related matrix associated, actin dependent regulator of chromatin, subfamily B, member 1
SOX10: SRY-box 10
ST18: Suppression of Tumorigenicity 18 zinc finger
TBR1: T-box brain 1
THBS1: Thrombospondin 1
XBP1: X-box binding protein 1
ZNRF3: Zinc and ring finger 3
