# Supplementary material for: Network Reconstruction Reveals that Valproic Acid Activates Neurogenic Transcriptional Programs in Adult Brain Following Traumatic Injury
Source: Pharm Res. 2017 Mar 7;34(8):1658–72. doi: 10.1007/s11095-017-2130-6 (PMC5498621; doi:10.1007/s11095-017-2130-6)
Supplement: Supplementary file 1 — (DOCX 2041 kb) [file 11095_2017_2130_MOESM1_ESM.docx]

**SUPPLEMENT**

**Network reconstruction reveals that valproic acid activates neurogenic transcriptional programs in adult brain following traumatic injury**

Gerald A. Higgins^1^, Patrick Georgoff^2^, Vahagn Nikolian^2^, Ari Allyn-Feuer^1^, Brian Pauls^3^,

Richard Higgins^4^, Brian D. Athey^1,5^, Hasan E. Alam^2^

^1^ Department of Computational Medicine and Bioinformatics, University of Michigan Medical School

^2^Department of Surgery, University of Michigan Medical School

^3^College of Pharmacy, University of Michigan

^4^Department of Computer Science, University of Maryland

^5^Michigan Institute for Data Science (MIDAS)

**Table of Contents**

**Supplementary Table 1:** Data used in the reconstruction of VPA’s transcriptional program in human brain**2**

**Supplementary Table 2.** VPA-master transcription factors expressed in human brain**4**

**Supplementary Figure 1:**  Reconstructed VPA regulatory network of TFs from IPA® **6**

**Supplementary Figure 2:** Reconstructed VPA regulatory network of TFs using STRING**7**

**Supplementary Table 3:** Reconstructed VPA pathway components and CNS cell fate**8**

**Supplementary Figure 3:** Gene set enrichment analysis (GSEA) of microarray expression data from swine brain following TBI and HS, followed by treatment with VPA**24**

**Supplementary Figure 4:** Gene set enrichment analysis of TFs, predicted in human brain**25**

**Supplementary Figure 5:** Spatial clustering of genes in the CNS VPA gene regulatory network**26**

**Supplementary Figure 6:** Estimated prevalence of VPA-responsive TFs **27**

**Supplemental References 28**

**Supplementary Table 1:** Data used in the reconstruction of VPA’s transcriptional program in human brain. See Figure 1. *NA*: Not available.

| **DATASET(S)** | **TYPE** | **NAME / LINK** | **REF** |
| --- | --- | --- | --- |
| Transcription factors (TFs) | Catalogues of known TFs, defined in text and characterized by family. | FactorBook: <http://factorbook.org> | **[S1]** |
|  |  | TransFac Pro® | **[S2]** |
| TF regulatory circuits | Interactive or downloadable SVG files describing nodes, edges, interactions and targets. | TF network browser:  <http://www.regulatorynetworks.org> | **[S3]** |
|  | Regulatory interactions in the human genome defined by *cis*- and *trans*-interactions. | Chromatin Chromatin Space Interaction:  <http://songyanglab.sysu.edu.cn/ccsi/search.php> | *NA* |
|  | Text description of TFs in human frontal cortex. |  | **[S4]** |
|  | Gene regulatory networks (GRNs) applied to stem cell engineering | <http://cellnet.hms.harvard.edu> | **[S5]** |
| TF promoter analysis | Detailed analysis of TF binding sites with genes and their promoters, as well as experimental data from ChIP-Seq studies. | TransFac Pro® (Qiagen GmbH) | **[S3]** |
| Genes regulated by TFs | Different types of TF-gene interaction analysis, including upstream and downstream regulators. | IPA® (Qiagen GmbH) | **[S6]** |
| Master TF super-enhancers with H3K27ac marks | Comprehensive ranking by super-enhancer of all human tissue-specific master TFs. | *Table S3.* Candidate Master Transcription Factors in 86 Human Samples | **[S5]** |
|  | *Same as above* | *Supplementary Information* | **[S7]**  **[S8]** |
| Master TFs | Classification of human TFs based on the characteristics of their DNA-binding domains. Comprises four general levels and two levels of instantiation. | TFClass:  <http://tfclass.bioinf.med.uni-goettingen.de> | **[S9]** |
|  | Spreadsheet containing lists of master TFs in different human tissues and cell lines. | *Supplementary Information* | **[S4]** |
| TF binding analysis | Catalog of PWM TF binding motifs | TransFac Pro® (Qiagen GmbH) | **[S3]** |
| Pioneer TFs that are CSRs | Comprehensive catalogue of human pioneer factors that remodel chromatin. | <https://github.com/dlampart/csrproject> | **[S10]** |
| HOT analysis | HOT (high-occupancy target) regions | *Supplemental Tables* providing information on distribution of HOT regions in human genome | **[S11]** |
| TF spatial contacts  SK-N-SH cells | Data from Hi-C and other chromatin capture methods to analyze genomewide spatial interactions. | 4DGenome:  <http://4dgenome.int-med.uiowa.edu> | **[S12]** |
|  | Experimental data that is unpublished. | *Proprietary experimental data*, Broad Institute | *NA* |
| Chromatin remodelers and HDACs | Compendium of proteins that participate in chromatin remodeling. | IPA® (Qiagen GmbH) | **[S6]** |
| TF and chromatin- mechanisms of cellular reprogramming | Description of conserved and novel network topology that is responsible for programming of cell fate during development, and/or TFs that can be used to reprogram cells into neurons. |  | **[S13]**  **[S14]**  **[S15]** |
| Experiments on VPA gene regulation in an animal model | Unpublished results from the laboratory of Dr. Alam. Several manuscripts are being prepared for submission. | *Unpublished datasets* | |

**Supplementary Table 2:** VPA- Master transcription factors in human brain

| **MASTER TFs*** | **TARGET TFs** |
| --- | --- |
| **ARID1A** | ARID1A, ARID1B, ARNT2, ASCL1, BCL6, BCL11A, BCL11B, BHLBHE40, BHLBHE41, CEBPA, CHAF1B, CUL2, CUL3, EGR1, ELK1, FOXA2, GATA1, GATA4, GFI1, HEY2, ID3, INO80, JUNB, KLF6, KLF9, KLF13, LHX2, MED1, MEF2C, MEF2D, MYT1L, NEUROD1, NFE2L2, NFIX, NKX2-2, NR1D1, NR3C1, NR4A1, NR4A2, NR6A1, OLIG2, PAX6, PLAG1, PPARD, POU3F2, POU3F3, POU5F1, PROX1, PRRX1, RARB, RBPJ, RXRA, SIRT1, SMARCA1, SMARCA2, SMARCB1, SMARCC1, SMARCC2, SMARCE1, SOZ2, SOX10, SPI1, SREBF1, ST18, TBR1, TCF4, TCF12, WNT1, ZHX2, ZNF24 |
| **ASCL1** | CDKN1C, CEBPA, DLX1, DLX2, DLX5, E2F1, ETV1, FOXM1, GATA3, GATA4, GATA6, GSX2, HAND1, HES1, HES5, HES6, HEY2, HNF4A, ID1, ID3, INSM1, ISL1, KLF4, LHX1, LHX5, LMX1B, MEF2A, MEF2C, MEF2D, MEIS1, MYC, NEUROD1, NEUROD2, NEUROD6, NEUROG2, NHLH2, NKX2-2, OLIG2, PAX1, PAX2, PAX3, PAX6, PAX8, PHOX2A, PHOX2B, POU4F2, POU5F1, PROX1, RBPJ, REST, RORB, SOX2, TBR1, TCF12, TCF3, TCF4, TEAD1, TEAD2, THRB, TLX1, TLX3, UNCX |
| **BCL11A** | BCL11A, BCL6, ETS1, GATA1, GATA3, IKZF1, KLF1, LIN28B, NR2E1, NR2E3, NR2F1, NR2F2, NR2F6, RCOR1, RUNX1, SPI1, STAT5A, TBR1, TFEC, TP53, YY1, ZBTB24, ZBTB33, ZFPM1 |
| **CHAF1B** | NFIX, NFKB1, NKX2-5, PPARA, PAX6, SMAD2, SMAD3, SMAD4, SMAD9, SOX2, TBX2, WT1 |
| **ELK1** | AR, CEBPA, EGR1, EGR2, ELK1, ELK3, ELK4, EMX1, ESR1, FLI1, FOS, FOSL1, GATA1, HLX, HOXB13, ID2, JUN, JUNB, KLF4, MECOM, NKX3-1, POU2F1, RUNX1, RUNX2, SNAI1, SP1, SRF, TCF4, ZHX2 |
| **MEF2C** | ARID1A, ASCL1, BHLHE40, BHLHE41, ESRRB, FOXJ3, FOXP1, GATA1, GATA2, GATA3, GATA4, GATA6, GLI1, GLI2, HAND1, HIF1A, JUN, KLF2, KLF4, MEF2A, MEF2C, MEF2D, MEOX1, MYOD1, MYOG, NFATC2, NFE2L2, NKX2-5, NR1D1, NR4A3, NRL PLAGL1, POU5F1, PPARA, RUNX2, SMAD2, SNAI2, SOX18, SOX2, SP1, SP7, SPI1, SPIB, SREBF1, TBX5, TCF3, TEAD1, TFCP2, TWIST2, ZNF335 |
| **MEF2D** | ASCL1, ESR1, FOS, GATA4, HAND1, IRF8, JUN, MEF2A, MEF2C MEF2D, MEOX2 MYOD1, MYOG, NFATC1, NFATC2, NR4A1, PPARA, SMAD3, SP1 |
| **NEUROD1** | ASCL1, CDX1, CDX2, CREB1, CRX, FOXA2, FOXN4, GFI1, GLIS3, HES1, ID1, ID3, INSM1, ISM2  ISL1, KLF9, LHX2, MAFA, MIXL1, MYLT1, NEUROD1, NEUROD2, NEUROG1, NEUROG2, NEUROG3, NFE2L2, NKX2-2, NKX6-1, NR1H4, NR4A1, NR4A2, NR4A3, NR6A1, PAX4, PAX6, PDX1, PITX1, POU3F2, POU4F1, POU4F2, RBPJ, REST, RREB1, SCT, SIX1, SP1, SREBF1, SREBF2, ST18, TCF12, TCF3, TCF4, TCF7L1, TLX3, ZFHX3 |
| **NR1D1** | ARNTL, ESR1, ESRRA, HNF1A, HNF4A, KLF6, MEF2C, NR1D1, NR1D2, PPARA, PPARG, RORA, RORC, RXRA, STAT1 |
| **NR6A1** | FOXA2, MYOG, NR6A1, POU5F1, TP53 |
| **PAX6** | BCL2L2, CUX2, EOMES, MITF, SHH, SIX6 |
| **POUF51** | PAX6 |
| **PPARD** | BCL6, CEBPA, CEBPB, EHF, ESR1, FOXO1, GATA3, HNF4A, KLF5, MEF2A, MYC, MYCL, NR1H3  ONECUT1, PPARA, PPARD, PPARG, PROX1, RELA, RUNX2, RXRA, RXRB, RXRG, SMAD3, SMAD9, SP1, SP7, STAT1, STAT3, TCF7, TFAP2A, TP53 |
| **PROX1** | ASCL1, ESRRA, ESRRG, ETS2, FOXC2, FOXN4, HEY1, HEY2, HNF1A, HNF4A, HOXD8, IRF4, MSX2, NR2F2, NR3C2, NR5A1, NR5A2, PAX6, POU4F2, PPARD, PRDM1, PROX1, RORA, SMAD3, SOX18, STAT6, YY1, ZBTB3 |
| **PRRX1** | ALX4, FOS, PAX7, PAX9, PGR, PRRX1, SOX2, SOX8, SOX9 |
| **SOX10** | ALX4, ARNT2, CEBPA, DLX5, EGR2, FOS, HES5, HHEX, HIVEP1, HNF4A, HOXA3, JUN, LHX1, MEOX1, MITF, OLIG2, PAX3, PAX6, POU3F2, POU3F3, SIM1, SNAI2, SOX10, SOX9, SP1, TCF12  TCF7L2 |
| **ST18** | FOXA2, MYT1, NEUROD1, NEUROG3, PDX1, ST18, TCF7L2 |
| **TBR1** | ASCL1, BCL11A, BCL11B, EOMES, PAX6, SATB2, TBR1, ZFHX3 |

*Note that many of the TFs are regulated by multiple TFs. Master TFs are defined on the basis of their regulation by super-enhancers **[S7, S8]**.

**Supplementary Figure 1:**  Reconstructed VPA regulatory network of TFs from IPA® **[S6]**

**
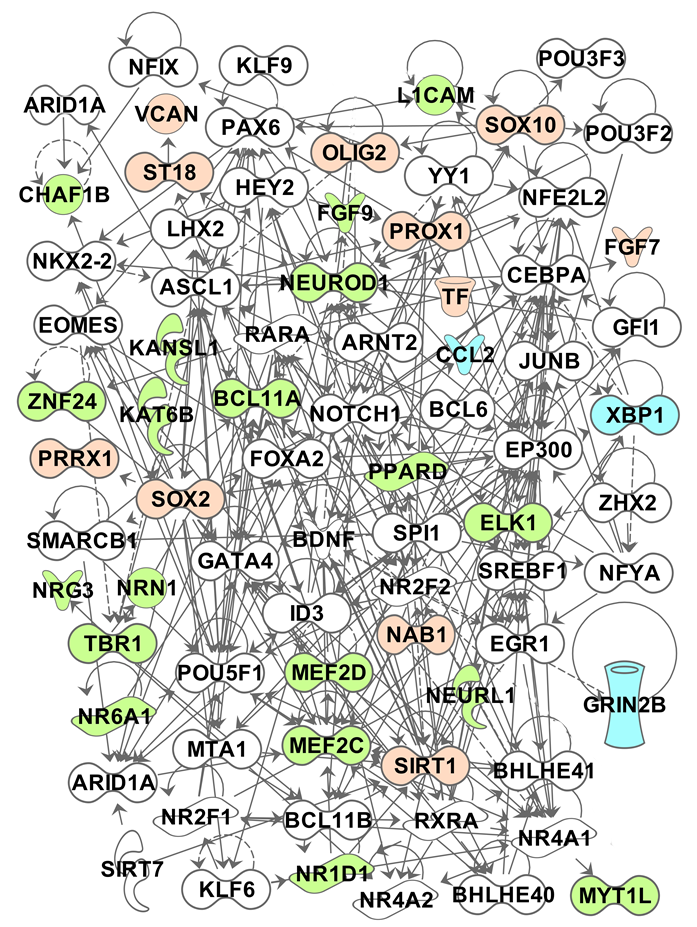
**

Master TFs are indicated in bold font. Yellow shading indicates genes that encode chromatin remodelers. Key:

**
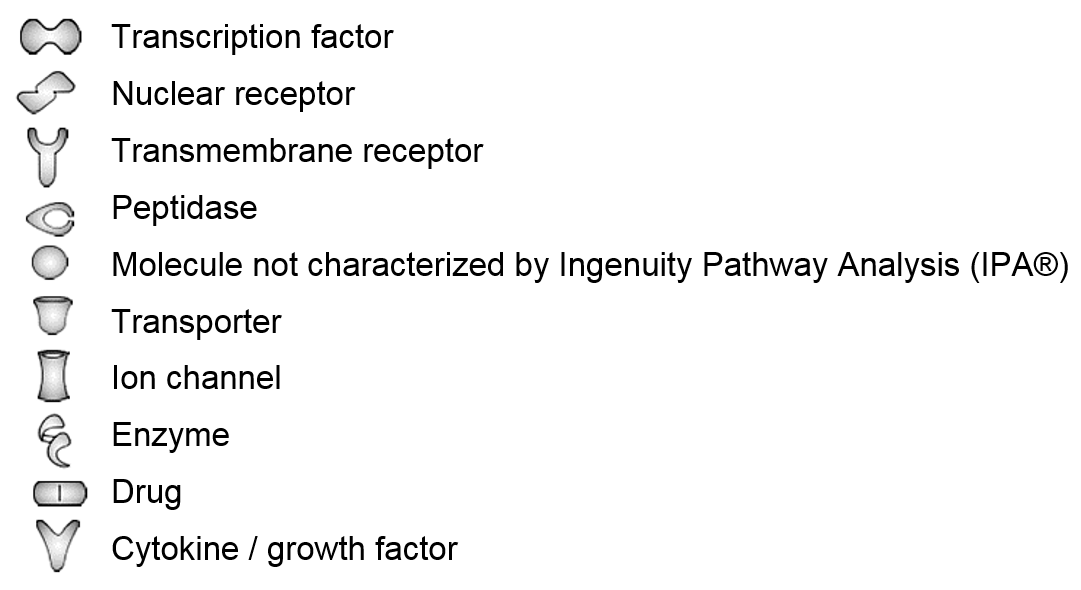
**

**Supplementary Figure 2:** VPA regulatory network reconstructed by STRING **[S16]**

**
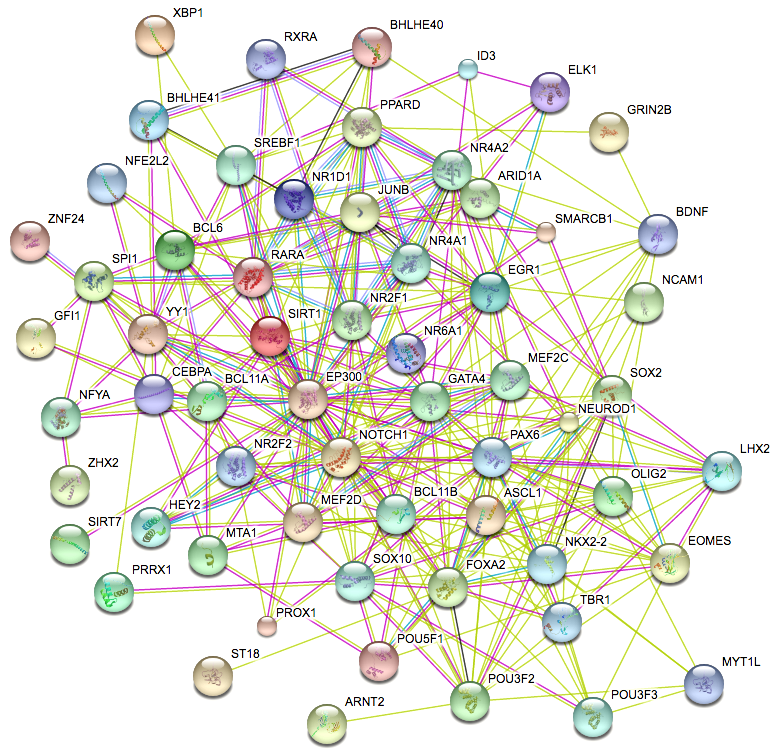
**

**Supplementary Table 3:** Reconstructed VPA pathway components and CNS cell fate

(Examples of selected attributes and publications, citations indicated by PubMed ID (PMID))

| **GENE** | **DEFINITION** | **DEVELOPMENT / TRANS-DIFFERENTIATION** | **PMID** |
| --- | --- | --- | --- |
| *ARID1A* | AT Rich Interactive Domain 1A (SWI-Like). Involved in transcriptional activation and repression of select genes by chromatin remodeling (alteration of DNA-nucleosome topology). Belongs to the neural progenitors-specific chromatin remodeling complex (npBAF complex) and the neuron-specific chromatin remodeling complex (nBAF complex). During neural development, a switch from a stem/progenitor to a post-mitotic chromatin remodeling mechanism occurs as neurons exit the cell cycle and become committed to their adult state. The npBAF complex is essential for the self-renewal/proliferative capacity of the multipotent neural stem cells. | - Switches neuronal cell fate as part of a chromatin remodeling complex; - ARID1A-DNA interactions are required for promoter occupancy by the chromatin remodeling complex SWI/SNF; - Controls subunit switching in the BAF (SWI/SNF) complex to determine neuronal cell fate; - Member of the neural progenitor-specific BAF (npBAF) complex that downregulates the pluripotency gene network promoted in ES cells while remaining proliferative, also suppress the neuronal differentiation program until mitotic exit; - Monoallelic loss of ARID1A in neural crest cells leads craniofacial defects in adult animal models; - Suppression of the ARID1A subunit of the SW/SNF complex promotes neural regeneration in adult mammals. | 17640523  23129809  23568486  25195934  26806701  27044474 |
| *ARNTL*  *(BMAL1)* | Aryl Hydrocarbon Receptor Nuclear Translocator-Like. Transcriptional activator which forms a core component of the circadian clock. The protein encoded by this gene is a basic helix-loop-helix protein that forms a heterodimer with CLOCK. This heterodimer binds E-box enhancer elements upstream of Period (PER1, PER2, PER3) and Cryptochrome (CRY1, CRY2) genes and activates transcription of these genes. | - Regulates neurogenic transcription factors, including NeuroD1, and the neuronal differentiation of adult neural stem/progenitor cells; - Control of circadian rhythm in early stages of neural progenitor cells; - Essential for neuronal fate commitment in the dentate gyrus of mice. | 19121353  25100653 26439128 |
| *ARNT2* | Aryl-Hydrocarbon Receptor Nuclear Translocator 2. This gene encodes a member of the basic-helix-loop-helix-Per-Arnt-Sim (bHLH-PAS) superfamily of transcription factors. | - Regulation of hypothalamic development; - Plays a role in the development of the hypothalamo-pituitary axis, postnatal brain growth, and visual and renal function; - Differentiation of hypothalamic-like neurons from human pluripotent stem cells. | 12947113 24022475  25428763  25555215 |
| *ASCL1*  *(MASH1)* | Achaete-Scute Family BHLH Transcription Factor 1. Member of the basic helix-loop-helix (BHLH) family. Controls transcriptional expression of its target genes by binding to the E box (5-CANNTG-3). Dimerization with other BHLH proteins is required for efficient DNA binding. Plays a role at early stages of development of specific neural lineages in most regions of the CNS, and of several lineages in the PNS. | - Regulates neurogenesis in the ventral telencephalon; - Specifies fate of human spinal cord neurons during development; - Direct conversion of fibroblasts to functional neurons; - Solely in control of the phase of expansion of neural progenitors and the subsequent phases of cell cycle exit and neuronal differentiation; - On its own, can convert human ESC’s to neurons; - In vivo reprogramming NG2 glia into neurons; - Conversion of neural stem cells (NSCs) located in the subventricular zone (SVZ) of the lateral ventricles into neurons. | 9876181  16715081  20107439  21536733  21617644  25254342  25827345  26730402 |
| *BCL6* | B-Cell CLL/Lymphoma 6. Zinc finger transcription factor and contains an N-terminal POZ domain. Transcriptional that forms complexes with different corepressors and histone deacetylases to repress the transcriptional expression of different subsets of target genes. | - Supports survival of hippocampal neurons through repression of NMDA-mediated excitotoxicity; - Conversion of neural stem/progenitor cells to neurons during CNS development with SIRT1; - Controls the transition from cortical progenitors to - pyramidal neurons; - Differentiation of human hippocampal granule neurons. | 17296556  23160044  24745669  25850787 |
| *BCL11A*  *(CTIP1)* | B-Cell CLL/Lymphoma 11A (Zinc Finger Protein). Plays a role in the control of microtubule-associated protein 1B in axonal growth and neuronal migration in the CNS. Regulated by multiple tissue-specific enhancers. | - Neuronal morphogenesis and sensory circuit formation in dorsal spinal cord development; - Helps with chromatin remodeling BAF complex in neural differentiation and reprogramming; - Specification of cortical sub-cerebral projection neurons through repression of TBR1; - Regulation of subtype identity in deep-layer projection neurons. | 22491945 25972180  27117402 |
| *BCL11B*  *(CTIP2)* | B-Cell CLL/Lymphoma 11B (Zinc Finger Protein). This gene encodes a C2H2-type zinc finger protein and is closely related to BCL11A. Functions as a novel, dedicated subunit of BAF, and acts in chromatin-mediated neurogenesis. | - Controls corticospinal motor neuron development in segment L5 only; - Controls the differentiation of medium spiny neurons and the establishment of the cellular architecture of the striatum; - Works with BCL11A in hippocampal neurogenesis. | 15664173  18199763  22588081 |
| *CEBPA* | CCAAT/Enhancer Binding Protein (C/EBP), Alpha. This intron-less gene encodes a transcription factor that contains a basic leucine zipper (bZIP) domain and recognizes the CCAAT motif in the promoters of target genes. Coordinates proliferation arrest and the differentiation of myeloid progenitors, adipocytes, hepatocytes, and cells of the lung and the placenta. | - In vitro reprogramming of fibroblasts and B cells into macrophages. | 18424555  20107439 |
| *CHAF1B* | Chromatin Assembly Factor 1, Subunit B (P60). Chromatin assembly factor I (CAF-I) is required for the assembly of histone octamers onto newly-replicated DNA. | - As part of the histone chaperone CAF-1 with CHAF1B, it prevents somatic cells from slipping back into ESC’s. - Suppression of CHAF1B led to a more accessible chromatin structure at enhancer elements early during reprogramming of neurons. | 20107439 |
| *EGR1* | Early Growth Response 1. The protein encoded by this gene belongs to the EGR family of C2H2-type zinc-finger proteins. It is a nuclear protein and functions as a transcriptional regulator, and targets genes that are required for differentiation and mitogenesis. | - Activated early in programmed cell death induced by trophic factor withdrawal from neuronal PC12 cells. - Implicated in neurogenesis during regulation by APE1 in a redox-dependent manner. | 7472489 24586617 |
| *ELK1* | ELK1, Member of ETS Oncogene Family. Binds to purine-rich DNA sequences and forms a ternary complex with SRF and the ETS and SRF motifs of the serum response element (SRE) on the promoter region of immediate early genes such as FOS and IER2 | - Directs glial cell differentiation in the embryonic CNS of *Drosophila*; - Controlled by mIR-143 in the regulation of vascular smooth muscle cell fate; - Serves as a key intermediate in drug-induced reprogramming in signaling pathways that bind and activate endogenous master neural genes - to specify neural identity. | 8033206  19578358  27133794 |
| *EOMES (TBR2)* | Eomesodermin. This gene belongs to the TBR1 (T-box brain protein 1) sub-family of T-box genes that share the common DNA-binding T-box domain. The encoded protein is a transcription factor which is crucial for embryonic development of the central nervous system in vertebrates. Required for the specification and the proliferation of the intermediate progenitor cells and their progeny in the cerebral cortex. | - Expressed sequentially with PAX6 by radial glia, intermediate progenitor cells, and postmitotic neurons in developing neocortex; - Regulates neurogenesis in the cortical subventricular zone; - Directs conversion of radial glia into basal precursors and guides neuronal amplification by indirect neurogenesis in the developing neocortex; - Directs lineage progression from neural stem cells to intermediate progenitors and neurons in hippocampus. - Directs cortical and clonal contribution neuronal progenitors in the developing mouse brain; - Regulates cell proliferation during neocortical development; - Reprogram any cell into neuron in culture. | 15634788  18794345  18940588  22553033 24927931  25191221  25512556 |
| *FOXA2* | Forkhead Box A2. Transcription factor that is involved in embryonic development, establishment of tissue-specific gene expression and regulation of gene expression in differentiated tissues. Acts as a pioneer factor opening the compacted chromatin for other proteins through interactions with nucleosomal core histones and thereby replacing linker histones at target enhancer and/or promoter sites. | - Directs conversion of neuronal progenitor cells into mesencephalic dopaminergic neurons in concert with LMX1A; - With FOXA1, it maintains dopaminergic properties in ventral midbrain neurons at late embryonic stages; - With the dimer CLOCK: ARNTL recruits H2A.Z in the role of a pioneer factor that targets promoters, promoting nucleosome loss prior to neuronal cell fate commitment. | 16439212  17670789  22813745  2363719  26826681 |
| *GATA4* | GATA Binding Protein 4. This gene encodes a member of the GATA family of zinc-finger transcription factors which recognizes the GATA motif which is present in the promoters of many genes. This protein is thought to regulate genes involved in embryogenesis, differentiation and cell-specific function. | - Epigenomic control of spontaneous hESC differentiation in presence of TF’s that specify neuronal cell fate; - Reprogramming of human fibroblasts to cardiac cells; - Induction of cardiac enhancers during directed conversion of fibroblasts to cardiomyocytes. | 18632693  23487791  23968684 |
| *GFI1* | Growth Factor Independent 1 Transcription Repressor. This gene encodes a nuclear zinc finger protein that functions as a transcriptional repressor. It functions as part of a complex along with other cofactors to suppress, via histone deacetylase (HDAC) recruitment, a number of genes involved in development. | - Interacts with PROX1 and ATOH1 to regulate cellular differentiation in the inner ear sensory epithelium; - Helps to regulate the development of parvalbumin-expressing cortical interneurons; - Directs reprogramming of progenitors to auditory hair cells in combination with POU4F3 and ATOH1. | 1865281520159447  26015538 |
| *HEY2* | Hes-Related Family BHLH Transcription Factor with YRPW Motif 2. A member of the hairy and enhancer of split-related (HESR) family of basic helix-loop-helix (bHLH)-type transcription factors. Localizes to the nucleus and interacts with a histone deacetylase complex to repress transcription. | - Contributes to determination of cell fate in the retina; - With Hey1 controls the spatial and temporal pattern of mammalian auditory hair cell differentiation; - Helps maintain cell fate in the cochlea. | 15036211  25232121  25593106 |
| *ID3* | Inhibitor Of DNA Binding 3, Dominant Negative Helix-Loop-Helix Protein. Transcriptional regulator (lacking a basic DNA binding domain) which negatively regulates the basic helix-loop-helix (bHLH) transcription factors by inhibiting their DNA binding and transcriptional activity. Regulates cellular growth and differentiation. | - Contributes to astroglial cell fate commitment from neural crest cells; - Causes detachment of cortical neural progenitor cells from radial glial fibers prior to cell migration; - With E47 determines neural stem/precursor cell differentiation into astrocytes. | 22489086  2397315826438726 |
| *INO80* | INO80 Complex Subunit. This gene encodes a subunit of the chromatin remodeling complex, which is classified into subfamilies depending on sequence features apart from the conserved ATPase domain. This protein is the catalytic ATPase subunit of the INO80 chromatin remodeling complex, which is characterized by a DNA-binding domain. | - Directs neuronal cell fate commitment from pluripotent cells in culture; - Differentiates mouse ESC’s into neuronal progenitor cells as part of chromatin remodeling complex; - Directs npBAF to nBAF programmed neuronal fate and converts fibroblasts into neurons; - Represses Bmp4 expression in the early embryo, thus promoting DVE differentiation and successful proximal-distal axis establishment. | 1732051124366184  25195934  26975355 |
| *JUNB* | Jun B Proto-Oncogene. Transcription factor involved in regulating gene activity following the primary growth factor response. Binds to the DNA sequence 5-TGA[CG]TCA-3. | - Helps in the regulation of proteins in the extracellular matrix to direct stem cells to CNS cell fate; - Controlled by functional neuronal activity-dependent enhancers during early CNS development. | 23839578  25195102 |
| *KLF6* | Kruppel-Like Factor 6. This gene encodes a member of the Kruppel-like family of transcription factors, and the zinc finger protein is a transcriptional activator. | - May help specify layer IV pyramidal neurons in mouse cerebral cortex; - Involved in axonal regeneration. | 15618518  23681442 |
| *KLF9* | Kruppel-Like Factor 9. Transcription factor that binds to GC box promoter elements. Selectively activates mRNA synthesis from genes containing tandem repeats of GC boxes but represses genes with a single GC box. | - Required for late-phase neuronal maturation in the developing dentate gyrus and during adult hippocampal neurogenesis; - Acts in pathway of thyroid hormone receptor for differentiation of pluripotent stem cells; - Inhibits glioblastoma stem cells through global transcription repression and integrin α6 inhibition; - Contributes to hippocampal neurogenesis. | 1965703925330987  25288800  26430216 |
| *KLF13* | Kruppel-Like Factor 13. Represses transcription by binding to the BTE site, a GC-rich DNA element, in competition with the activator SP1. It also represses transcription by interacting with the corepressor SIN3A and HDAC1. | - Represses histone deacetylase inhibition-mediated neuronal differentiation of multipotent adult neural progenitor cells; - Pro-apoptotic activity enhances conversion of progenitor cells to neurons. | 23881454  26027682 |
| *LHX2* | LIM Homeobox 2. Acts as a transcriptional activator. Transcriptional regulatory protein involved in the control of cell differentiation in developing neural cell types. | - Necessary and sufficient to suppress astrogliogenesis and promote neurogenesis in the developing hippocampus; - Regulates corticogenesis in mice through progenitor differentiation; - Specifies maturation of retinal progenitor cells; - Neuronal subtype specification in establishing mammalian neocortical circuits; - Direct conversion of adult mouse liver cells and B lymphocytes to neural stem cells; - Controls switch from progenitor to mature neurons in cerebral cortex. | 21690374 23454273 2388492825019611  25454632  26321900 |
| *MEF2C* | Myocyte Enhancer Factor 2C. Plays an essential role in hippocampal-dependent learning and memory by suppressing the number of excitatory synapses and thus regulating basal and evoked synaptic transmission. Crucial for normal neuronal development, distribution, and electrical activity in the neocortex. | - Direct conversion of fibroblasts into 'cardiomyocyte-like cells' - Specifies striatonigral cell fate in mouse brain; - Direct conversion of endogenous neural progenitor cells in the cerebral cortex to neurons; | 20691899  24351932  24942075 |
| *MEF2D* | Myocyte Enhancer Factor 2D. This gene is a member of the myocyte-specific enhancer factor 2 (MEF2) family of transcription factors. Members of this family are involved in control of neuronal cell differentiation and development, and are regulated by class II histone deacetylases. | - Phosphorylation of MEF2D promotes neuronal survival after DNA damage in human CNS; - Suppresses IL-10 production in microglia to protect neuronal cells from inflammation-induced death; - Directs photoreceptor development through a genome-wide competition for tissue-specific enhancers; - Activation protects dopaminergic neurons and ameliorates Parkinsonian motor defects; | 22891246 24672010  25801704  25890150 |
| *MYT1L* | Myelin Transcription Factor 1-Like. A pan-neural transcription factor associated with neuronal differentiation. Plays a role in the development of neurons and oligodendroglia in the CNS. | - Direct conversion of fibroblasts to functional neurons; - Direct reprogramming of astrocytes and fibroblasts into neurons following transplantation; - Direct conversion of fibroblasts to medium spiny neurons; - Direct conversion of fibroblasts to motor neurons; - Direct conversion of fibroblasts to dopaminergic neurons. | 20107439  21617644  2353023526475975 |
| *NEUROD1* | Neuronal Differentiation 1. A transcriptional activator that binds to the E box-containing promoter consensus core sequences 5-CANNTG-3. Associates with the p300/CBP transcription coactivator complex to stimulate transcription. Contributes to the regulation of several cell differentiation pathways, like those that promote the formation of early retinal ganglion cells, inner ear sensory neurons, granule cells forming the cerebellum and the dentate gyrus cell layer of the hippocampus. | - Specifies hippocampal cell fate from neuroblasts; - In vivo direct reprogramming of reactive glial cells into functional neurons after brain injury; - Induces terminal neuronal differentiation in olfactory neurogenesis; - Conversion of astrocytes to neurons by releasing REST suppression; - Directs neurogenesis and integration of new neurons into functional systems after spinal cord injury; - in vivo can directly reprogram reactive glial cells into glutamatergic and GABAergic neurons that integrate into the host's neural circuitry after brain injury; - Direct reprogramming of any cell type that has been tested into mature neurons; - Master regulator that reprograms chromatin and transcription factor landscapes to induce the neuronal program in all cells studied to date; - Acts as pioneer factor to alters chromatin state to drive neuronal cell fate in all cells studied to date; | 19274100  20080708  24360883  24771471  24506877  25554728  26140600  26119235  26416679  26516211  26567170 |
| *NFE2L2* | Nuclear Factor, Erythroid 2-Like 2. This gene encodes a transcription factor which is a member of a small family of basic leucine zipper (bZIP) proteins. Transcription activator that binds to antioxidant response (ARE) elements in the promoter regions of target genes. | - Directs cell fate conversion of different cell types during neurodegenerative disease; - Prevents oxidative-induced cell death of retinal ganglion cells; - promotes neuronal survival in neurodegeneration and acute nerve damage | 231262262372154625798616 |
| *NFIX* | Nuclear Factor I/X (CCAAT-Binding Transcription Factor). Recognizes and binds the palindromic sequence 5-TTGGCNNNNNGCCAA-3 present in cellular promoters and in the origin of replication of adenovirus type 2. These proteins are individually capable of activating transcription and replication. | - Regulates neural progenitor cell differentiation during hippocampal morphogenesis in rodent brain; - Knockout biases postnatal neural stem/progenitor cells toward oligodendrogenesis; - Recapitulate gene expression programs of fetal neocortex development | 23042739  26083238  26644564 |
| *NKX2-2* | NK2 Homeobox 2. The protein encoded by this gene contains a homeobox domain involved in the morphogenesis of the central nervous system. Transcriptional activator that associates with chromatin at the NEUROD1 promoter region and binds to a subset of consensus elements within the NEUROD1 promoter. Involved in specifying diencephalic neuromeric boundaries, and in controlling the expression of genes that play a role in axonal guidance. | - Specifies neuronal identity by graded Sonic hedgehog signaling in CNS; - Control of cortical interneuron cell fate; - Key regulator that determines cell fate of branchial and visceral motor neurons in caudal hindbrain. | 1021714524440413  25919494 |
| *NOTCH1* | Notch 1. This gene encodes a member of the Notch family. Members of this Type 1 transmembrane protein family share structural characteristics including an extracellular domain consisting of multiple epidermal growth factor-like (EGF) repeats, and an intracellular domain consisting of multiple, different domain types. Plays a role in a variety of developmental processes by controlling cell fate decisions. | - In animal models, mutations in *NOCTH1* produce an excess of neurons during development; - In breast, drives stem cells and progenitor cells towards a myoepithelial cell fate; - Direct conversion of fibroblasts into neural progenitor cells that can differentiate into neurons, astrocytes, and oligodendrocytes which all share genome-wide gene expression patterns and enhancer usage as primary-derived control neural stem cell lines; - Modulates the responsiveness of neural progenitors to PROX1-regulated sonic hedgehog signaling. | 14973298  15535842  25936505 |
| *NR1D1* | Nuclear Receptor Subfamily 1, Group D, Member 1. Transcriptional repressor which coordinates circadian rhythm and metabolic pathways. Integral component of the complex transcription machinery that governs circadian rhythmicity and forms a critical negative limb of the circadian clock by directly repressing the expression of core clock components ARTNL/BMAL1, CLOCK and CRY1. | - Regulate neural outgrowth in developing CNS; - Directed differentiation of neuronal cell fate; - Helps in the patterning, specification, and differentiation of the developing hypothalamus. | 23219993  25977693  25820448 |
| *NR2F1*  *(COUP-TF1)* | Nuclear Receptor Subfamily 2, Group F, Member 1. The protein encoded by this gene is a nuclear hormone receptor and transcriptional regulator. The encoded protein acts as a homodimer and binds to 5'-AGGTCA-3' repeats. | - Corticogenesis from embryonic stem cells; - Differentiation of glial cells in developing CNS. | 18716623  23840004 |
| *NR2F2*  *(COUP-TF2)* | Nuclear Receptor Subfamily 2, Group F, Member 2. Ligand-activated transcription factor. Activated by high concentrations of 9-cis-retinoic acid and all-trans-retinoic acid, but not by dexamethasone, cortisol or progesterone. | - Specifies regional neuronal organization of human neocortex; - Corticogenesis from embryonic stem cells; - First step in neural induction of human ESC’s; - Promotes radial migration and proper morphology of callosal projection neurons. | 18524571  18716623  21151097  21965613 |
| *NR4A1*  *(NUR77)* | Nuclear Receptor Subfamily 4, Group A, Member 1. This gene encodes an orphan nuclear receptor that is member of the steroid-thyroid hormone-retinoid receptor superfamily. | - Specifies regional neuronal organization of human neocortex; - Corticogenesis from embryonic stem cells; - First step in neural induction of human ESC’s; - Promotes radial migration and proper morphology of callosal projection neurons. | 18524571  18716623  21151097  21965613 |
| *NR4A2*  *(NURR1)* | Nuclear Receptor Subfamily 4, Group A, Member 2. Transcriptional regulator which is important for the differentiation and maintenance of meso-diencephalic dopaminergic neurons during development. | - Directs terminal fate, somatic stem cell and in vivo reprogramming of stem cells into neurons; - Essential for development of midline dopaminergic cells; - Directed conversion of mouse fibroblasts into neurons in culture. | 24434846255653532642488626475975 |
| *NR6A1*  *(GCNF)* | Nuclear Receptor Subfamily 6, Group A, Member 1. This gene encodes an orphan nuclear receptor which is a member of the nuclear hormone receptor family, important for neurogenesis. | - Knockout of NR6A1 in a mouse embryonal carcinoma cell line showed that was critical for differentiation and maturation of neuronal precursor cells; - Required for the repression of pluripotency genes during retinoic acid-induced embryonic stem cell differentiation into neurons; - Master stem cell transcription factor; - Required for neurulation and specification of anterior-posterior axis of CNS in several species; - Repressor of POU5F1 (OCT4) gene expression during the epiblast-neural plate stages of embryonic development in mammals; - Activation of the core pluripotency circuitry. | 15297607  16166633  20132009  22634835  22992956  23681063 |
| *OLIG2* | Oligodendrocyte Lineage Transcription Factor 2. Required for oligodendrocyte and motor neuron specification in the spinal cord, as well as for the development of somatic motor neurons in the hindbrain. Cooperates with OLIG1 to establish the pMN domain of the embryonic neural tube. Antagonist of V2 interneuron and of NKX2-2-induced V3 interneuron development. | - Subcellular localization following brain injury determines glial cell fate; - Down-regulated when NG2-expressing cells (NG2 cells or polydendrocytes) cell fate is directed astrocytes instead of oligodendrocytes; - Overexpression pushes precursor cells in hippocampal cell culture to early maturation and increases the frequency of oligodendrocyte phenotypes - Direct conversion of fibroblasts into oligodendrocyte progenitor cells in mice; - Determines oligodendrocyte cell fate; - HDAC3 and STAT3 compete for EP300 for activation of oligodendrocyte cell fate; - Regulated by POU3F3 (BRN1) for oligodendrocyte progenitor cell identity. | 19473238 22627280  22162276  26475975  26859354  27067865 |
| *PAX6* | Paired Box 6. Encodes a homeobox and paired domain-containing protein that binds DNA and functions as a regulator of transcription. transcription factor with important functions in the development of the eye, nose, central nervous system and pancreas. Regulates specification of ventral neuron subtypes by establishing the correct progenitor domains. | - Overexpression pushes neuronal precursor cells in hippocampal cell culture to early maturation and increases the frequency of neuronal phenotypes; - Interacts with the BAF complex containing ARID1A and SMARCB1 in adult neuronal progenitors to establish a neurogenic cross-regulatory transcriptional network; - Essential for the maintenance and multi-lineage differentiation of neural stem cells; - Master regulator of corticogenesis acting as a pioneer factor with the chromatin remodeling complex BAF; - Directly regulates cell cycle exit into neurons via components of PROX1 and WNT signaling pathways; - Mutations in *PAX6* in humans significantly reduce frontoparietal cortex volume concomitant with severe working memory deficits. | 2216227623933087  25117830  25805971  26138486  27231702 |
| *POU3F2*  *(BRN2)* | POU Class 3 Homeobox 2. This gene encodes a member of the POU-III class of neural transcription factors. The encoded protein is involved in neuronal differentiation and enhances the activation of corticotropin-releasing hormone regulated genes. | - Direct conversion of fibroblasts to functional neurons; - Involved in upper-layer neuronal migration and identification, playing overlapping roles with POUF3F3 in the regulation of neocortical layers in development; - Direct conversion of adult mouse liver and B lymphocytes into neural stem cells; | 11859196  12130536  20107439  2161764425454632 |
| *POU3F3*  *(BRN1)* | POU Class 3 Homeobox 3. Transcription factor that plays a role in neuronal development. Is implicated in an enhancer activity at the embryonic met-mesencephalic junction; the enhancer element contains the octamer motif (5-ATTTGCAT-3). | - Involved in upper-layer neuronal migration and identification, playing overlapping roles with POUF3F2 in the regulation of neocortical layers in development; - Activates OLIG2 for oligodendrocyte progenitor cell identity. | 11859196  12130536  27067865 |
| *POU5F1*  *(OCT4)* | POU Class 5 Homeobox 1. This gene encodes a transcription factor containing a POU homeodomain that plays a key role in embryonic development and stem cell pluripotency. | - Regulation of chromatin structure in a state consistent with self-renewal and pluripotency; - Resetting the epigenome of a somatic cell to a pluripotent state using somatic cell nuclear transfer; - Activation of the core pluripotency circuitry. | 17579724  23681063  25482558 |
| *PROX1* | Prospero Homeobox 1. Transcription factor involved in developmental processes such as cell fate determination, gene transcriptional regulation, and progenitor cell regulation. Plays a critical role in embryonic development and functions as a key regulatory protein in neurogenesis and CNS development. Involved in the regulation of the circadian rhythm. | - Interacts with GFI1 and ATOH1 to regulate cellular differentiation in the inner ear sensory epithelium; - After mitosis, specifies granule cell identity versus pyramidal cell fate in hippocampus; - Regulates olig2 expression to modulate binary fate decisions in spinal cord neurons. - Determination of GABAergic cortical interneuron cell fate. | 18652815  22791897  25411508  26377473 |
| *PRRX1* | Paired Related Homeobox 1. The DNA-associated protein encoded by this gene is a member of the paired family of homeobox proteins localized to the nucleus. The protein functions as a transcription co-activator required for the induction of genes by growth and differentiation factors. | - Blocks neuronal cell death following ischemic injury; - Maintains pluripotency of adult neural stem/progenitor cells; - Can direct conversion of a neural stem cell to an oligodendrocyte progenitor cell in humans; - Partners with SOX2 to regulate neural stem cell fate. | 2342950623447615  24982138  25258670 |
| *RBPJ* | Recombination Signal Binding Protein For Immunoglobulin Kappa J Region. Transcriptional regulator that plays a central role in Notch signaling, a signaling pathway involved in cell-cell communication that regulates a broad spectrum of cell-fate determinations. Represses or activates transcription via the recruitment of chromatin remodeling complexes containing histone deacetylase or histone acetylase proteins. | - Suppresses glutamatergic and promotes GABAergic neuronal fate in the dorsal neural tube; - Regulates development of neuronal sensory cells in the mammalian inner ear; - Regulates NOTCH1 signaling in hypothalamus for neuronal differentiation in the arcuate nucleus; | 24370451  25593106  26318021 |
| *RXRA* | Retinoid X Receptor, Alpha. Retinoid X receptors (RXRs) and retinoic acid receptors (RARs) are nuclear receptors that mediate the biological effects of retinoids by their involvement in retinoic acid-mediated gene activation. These receptors function as transcription factors by binding as homodimers or heterodimers to specific sequences in the promoters of target genes. | - Acts as a transcriptional c0-regulator during differentiation of neural crest cells; - Maintains stem/progenitor cell pluripotency during embryonic development - Drug-induced neuronal differentiation and neural plasticity, including dendritic arborization. | 229909922523314126311769 |
| *SIRT1* | Sirtuin 1. NAD-dependent protein deacetylase that links transcriptional regulation directly to intracellular energetics and participates in the coordination of several separated cellular functions. Can modulate chromatin function through deacetylation of histones and can promote alterations in the methylation of histones and DNA, leading to transcriptional repression. Deacetylates a broad range of transcription factors and co-regulators. | - Epigenomic programming of ESC’s to neuronal progenitor cells; - Inactivation leads to proliferation of oligodendrocyte progenitor cells in the adult brain; - Controls neurogenic potential of neuronal precursor cells in the subventricular zone in the adult brain; - Conversion of neural stem/progenitor cells to neurons during CNS development; - Drives the differentiation of neuronal stem cells from pluripotent ESCs and iPSCs. | 19007844  23644469  23404532  25850787 |
| *SMARCB1*  *(SFN5)* | SWI/SNF Related, Matrix Associated, Actin Dependent Regulator Of Chromatin, Subfamily B, Member 1. Core component of the BAF (hSWI/SNF) complex. This ATP-dependent chromatin-remodeling complex plays important roles in neuron proliferation and differentiation. Belongs to the neural progenitors-specific chromatin remodeling complex (npBAF complex) and the neuron-specific chromatin remodeling complex (nBAF complex). During neural development a switch from a stem/progenitor to a post-mitotic chromatin remodeling mechanism occurs as neurons exit the cell cycle and become committed to their adult state. The transition from proliferating neural stem/progenitor cells to post-mitotic neurons requires a switch in subunit composition of the npBAF and nBAF complexes. | - In *C. Elegans* and *Drosophila*, deletion of the SMARCB1 homolog results in loss of primary dendrite arborization and other CNS defects; - Mice that lack Smarcb1 die in pre- or peri-implantation stages and/or exhibit microcephaly. | 1764052319442513 |
| *SOX2* | SRY (Sex Determining Region Y)-Box 2. This intron-less gene encodes a member of the SRY-related HMG-box (SOX) family of transcription factors involved in the regulation of embryonic development and in the determination of cell fate. Keeps neural cells undifferentiated by counteracting the activity of proneural proteins and suppresses neuronal differentiation. | - Marks active astrocytes (NSCs) and neural progenitors of the subventricular zone of progenitor cells in adult rodent brain; - Acts in a dose-dependent fashion to regulate proliferation of cortical progenitors; - Resetting the epigenome of a somatic cell to a pluripotent state using somatic cell nuclear transfer; - Activation of the core pluripotency circuitry; - *In vivo* reprogramming NG2 glia into neurons; - Directs pluripotent epigenetic landscapes via interactions with histone H2A.Z, recruiting polycomb repressor complex 2 in neural progenitor cells. | 9185542  12514105  23681063  25482558  26730402  26809499 |
| *SOX10* | SRY (Sex Determining Region Y)-Box 10. This gene encodes a member of the SOX (SRY-related HMG-box) family of transcription factors involved in the regulation of embryonic development and in the determination of the cell fate. Confers cell specificity to the function of other transcription factors in developing and mature glia. | - Genesis of neural crest-like cells from all dorsoventral levels of the neural tube but not differentiation; - Direct conversion of oligodendrocytes to neurons following injury in a rodent model; - Development of human enteric nervous system; - Directly induces differentiation of oligodendrocytes, including OLIGO1 and OLIGO2. | 15768395  22173870  23639815  25680202 |
| *SPI1*  *(PU.1)* | Spi-1 Proto-Oncogene. The nuclear protein binds to a purine-rich sequence known as the PU-box found near the promoters of target genes, and regulates their expression in coordination with other transcription factors and cofactors. | - Modulates early lineage decisions of hematopoietic progenitors towards either an erythrocyte and megakaryocyte or a granulocyte and monocyte fate; - Direct conversion of adult mouse liver and B lymphocytes into neural stem cells. | 18371378  25454632 |
| *ST18* | Suppression Of Tumorigenicity 18, Zinc Finger. Repressor that binds to DNA sequences containing a bipartite element consisting of a direct repeat of the sequence 5-AAAGTTT-3 separated by 2-9 nucleotides. Represses basal transcription activity from target promoters. | - Direct conversion of mouse fibroblasts into oligodendrocyte precursor cells. | 23584611 |
| *TBR1* | T-Box, Brain, 1. This gene is a member of a conserved family of genes that share a common DNA-binding domain, the T-box. T-box genes encode transcription factors involved in the regulation of developmental processes. A similar protein has been disrupted in mice and shown to be critical for early cortical development, and causes loss of projection neurons in the olfactory bulbs and olfactory cortex. | - Cooperates with Sox5 to regulate early born neurons in multiple lines during the embryonic development; - Cooperates with FEZF2 to regulate alternate corticofugal neuronal identities during neocortical development; - Regulates regional and laminar identity of post-mitotic neurons in developing neocortex. | 11239428  20615956  21228164 |
| *TCF4* | Transcription Factor 4. This gene encodes transcription factor 4, a basic helix-loop-helix transcription factor. The encoded protein recognizes an E-box binding site ('CANNTG'). This gene is broadly expressed, and may play an important role in nervous system development. | - Double knockout mice exhibit disrupted pontine nucleus; - Silencing of human TCF4 in cell lines affects multiple signaling pathways involved in cell survival and neuronal differentiation; - With PLAGL1, responsible for cell cycle arrest during neuronal progenitor differentiation; | 17878293 2405841424396065 |
| *ZHX2* | Zinc Fingers and Homeoboxes 2. The members of the zinc fingers and homeoboxes gene family are nuclear homodimeric transcriptional repressors that interact with the A subunit of nuclear Factor-Y (NF-YA) and contain two C2H2-type zinc fingers and five homeobox DNA-binding domains. | - Helps maintain neural progenitor in developing cerebral cortex; - Neural progenitor cell-specific protein that shapes methylation architecture in developing brain. | 19515908  23698584 |
| *ZNF24* | Zinc Finger Protein 24. Transcription factor required for myelination of differentiated oligodendrocytes. Required for the conversion of oligodendrocytes from the pre-myelinating to the myelinating state. In the developing CNS, involved in the maintenance in the progenitor stage by promoting the cell cycle. | - Chromatin regulation during switch to myelination state in oligodendrocytes. | 25970296 |

**Supplementary Figure 3.** Gene set enrichment analysis (GSEA) of microarray expression data from swine brain following TBI and HS, followed by treatment with VPA

**A B**


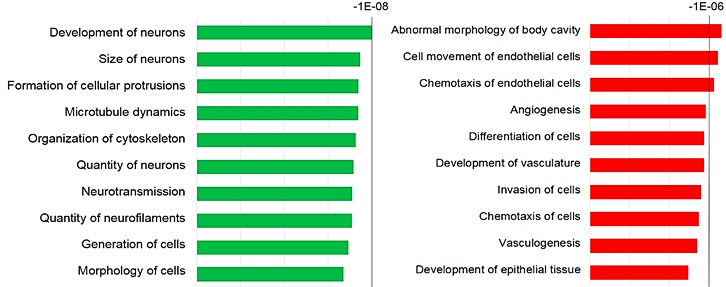


**Supplementary Figure 3.** Gene set enrichment analysis (GSEA) of microarray expression data from swine brain following TBI and HS, followed by treatment with VPA. **(A)** The 50 top up-regulated and **(B)** The 50 most down-regulated genes in brain. Significance determined using Fisher’s exact test as determined by the “grow” function in IPA® assessing function, but not disease. GSEA results in IPA® are corrected for multiple testing using Benjamini and Hochberg’s false discovery rate (FDR) **(**see **(42)** in main text**).**

**Supplementary Figure 4:** Gene set enrichment analysis of TFs, predicted in human brain


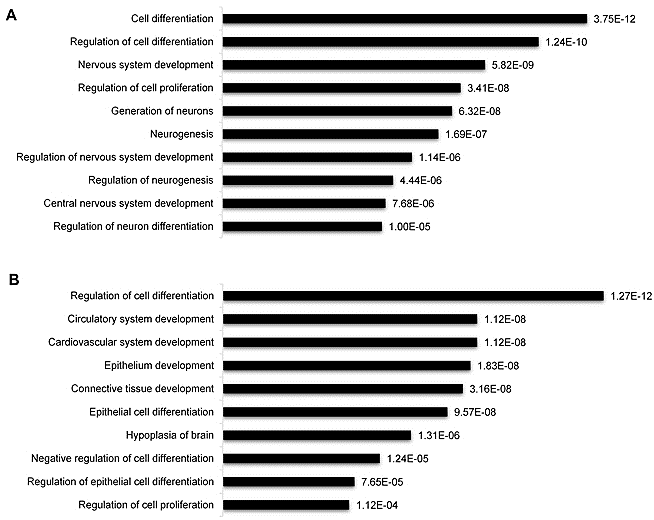


**Supplementary Figure 4. (A)** VPA-responsive TFs, and **(B)** TFs not regulated by VPA in adult brain, based on pre-existing data. Both Gene Ontology and IPA® were used for this analysis, using matching of ontology hierarchies.

**Supplementary Figure 5:** Spatial clustering of genes in the CNS VPA gene regulatory network

The data-driven strategy for gene network reconstruction integrated dissimilar data types from different sources **(Figure 1; Table 1)**, but consistently produced the same results. Supplementary Figure 4 show co-expression clusters within the swine brain injury dataset. The purpose of this analysis is to identify groupings of genes that co-express (either up or down regulated) because of the VPA treatment. The gene network is analyzed by raising co-expression values between genes to a power and identifying the power that correlates to biologically relevant gene clustering. The results demonstrate hierarchical, topogically-organized gene clusters following VPA treatment versus disorganization following CNS injury without VPA therapy.

**
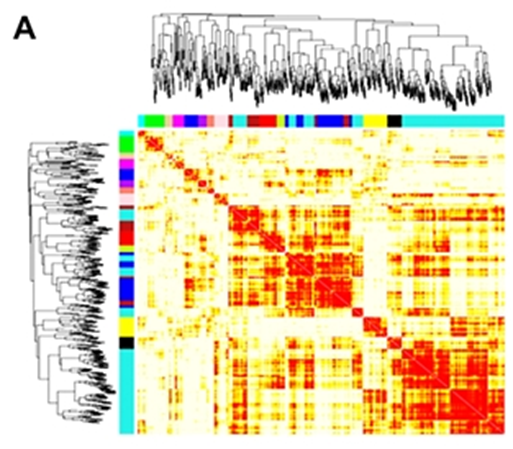

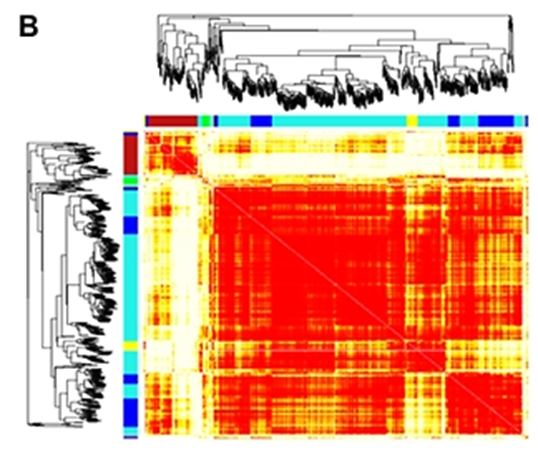
**

**Supplementary Figure 5. Heat-maps generated to compare the organization of gene co-expression clustering following VPA treatment. (A)** VPA and **(B)** vehicle treatment produce very different results. The VPA has several modules that contain topologically-organized sets of gene clusters, while vehicle treatment genes are grouped together. This suggests a lack of circumscribed topology of gene expression based on known organizational attributes.

**Supplementary Figure 6:** Estimated prevalence of VPA-responsive TFs

**Supplementary Figure 6. Estimated prevalence of VPA-responsive TFs, as percentage of all known TFs in humans, expressed in human telencephalon and during neurogenesis in developing CNS.** Included are estimates of the percentage of all VPA located in HOT regions of the human genome and those which are targeted by ARID1A, a member of chromatin remodeling complexes responsible for neurogenesis.

**SUPPLEMENT REFERENCES**

**[S1**] Wang J et al. Factorbook. org: A Wiki-based database for transcription factor-binding data generated by the ENCODE consortium. Nucleic Acids Res. 2013. (D1), D171-6.

**[S2]** Wingender E. The TRANSFAC project as an example of framework technology that supports the analysis of genomic regulation. Briefings in bioinformatics 2008;9:326-32.

**[S3]** Marbach D, Lamparter D, Quon G, Kellis M, Kutalik Z, Bergmann S. Tissue-specific regulatory circuits reveal variable modular perturbations across complex diseases. Nature methods. 2016 Mar 7.

**[S4]** Neph S et al. Circuitry and dynamics of human transcription factor regulatory networks. Cell. 2012. 150(6), 1274-1286.

**[S5]** Berto S, Perdomo-Sabogal A, Gerighausen D, Qin J, Nowick K. A consensus network of gene regulatory factors in the human frontal lobe. Frontiers in Genetics. 2016;7.

**[S6]** Kramer A, Green J, Pollard J, Jr., Tugendreich S. Causal analysis approaches in Ingenuity Pathway Analysis. Bioinformatics (Oxford, England) 2014;30:523-30.

**[S7]** Hnisz D, Abraham BJ, Lee TI, Lau A, Saint-André V, Sigova AA, Hoke HA, Young RA. Super-enhancers in the control of cell identity and disease. Cell. 2013 Nov 7;155(4):934-47.

**[S8]** Whyte WA, Orlando DA, Hnisz D, Abraham BJ, Lin CY, Kagey MH, Rahl PB, Lee TI, Young RA. Master transcription factors and mediator establish super-enhancers at key cell identity genes. Cell. 2013 Apr 11;153(2):307-19.

**[S9]** Wingender, E., Schoeps, T. and Dönitz, J. TFClass: An expandable hierarchical classification of human transcription factors. Nucleic Acids Res. 41, D165-D170 (2013).

**[S10]** Lamparter DF, Marbach D, Rueedi R, Bergmann S, Kutalik Z. Genome-wide association between transcription factor expression and chromatin accessibility reveals chromatin state regulators. bioRxiv. 2016 Jan 1:043414.

**[S11]** Li H, Liu F, Ren C, Bo X, Shu W. Genome-wide identification and characterization of HOT regions in the human genome. bioRxiv preprint first posted online Jan. 7, 2016; doi: http://dx.doi.org/10.1101/036152

**[S12]** Teng L et al. 4DGenome: a comprehensive database of chromatin interactions. Bioinformatics. 2015. 31(15), 2560-2564.

**[S13]** Zaret KS, Mango SE. Pioneer transcription factors, chromatin dynamics, and cell fate control. Current opinion in genetics & development. 2016 Apr 30;37:76-81.

**[S14]** Rackham OJ, Firas J, Fang H, Oates ME, Holmes ML, Knaupp AS, Suzuki H, Nefzger CM, Daub CO, Shin JW, Petretto E. A predictive computational framework for direct reprogramming between human cell types. Nature Genetics. 2016 Jan 18.

**[S15]** Gopalakrishnan S, Hor P, Ichida JK. New approaches for direct conversion of patient fibroblasts into neural cells. Brain research. 2015 Oct 16.

**[S16]** Szklarczyk D, Franceschini A, Wyder S, Forslund K, Heller D, Huerta-Cepas J, Simonovic M, Roth A, Santos A, Tsafou KP, Kuhn M. STRING v10: protein–protein interaction networks, integrated over the tree of life. Nucleic acids research. 2014 Oct 28:gku1003.

**[S17]** Mi H, Poudel S, Muruganujan A, Casagrande JT, Thomas PD. PANTHER version 10: expanded protein families and functions, and analysis tools. Nucleic Acids Research. 2016 Jan 4;44(D1):D336-42.
